# Wild *Helianthus* species: A reservoir of resistance genes for sustainable pyramidal resistance to broomrape in sunflower

**DOI:** 10.3389/fpls.2022.1038684

**Published:** 2022-10-20

**Authors:** Mireille Chabaud, Marie-Christine Auriac, Marie-Claude Boniface, Sabine Delgrange, Tifaine Folletti, Marie-Françoise Jardinaud, Alexandra Legendre, Begoña Pérez-Vich, Jean-Bernard Pouvreau, Leonardo Velasco, Philippe Delavault, Stéphane Muños

**Affiliations:** ^1^ Laboratoire des Interactions Plantes-Microbes- Environnement (LIPME), Université de Toulouse, Institut National de Recherche pour l'Agriculture, l'Alimentation et l'Environnement (INRAE), Centre National de la Recherche Scientifique (CNRS), Castanet-tolosan, France; ^2^ Unité en Sciences Biologiques et Biotechnologies (US2B), Nantes Université, Centre national de la recherche scientifique (CNRS), Unité mixte de recherche 6286 (UMR 6286), Nantes, France; ^3^ IAS-CSIC, Córdoba, Spain

**Keywords:** *Helianthus*, *Orobanche cumana*, sunflower, genetic resistance, phenotyping, cytology

## Abstract

*Orobanche cumana* Wall., sunflower broomrape, is one of the major pests for the sunflower crop. Breeding for resistant varieties in sunflower has been the most efficient method to control this parasitic weed. However, more virulent broomrape populations continuously emerge by overcoming genetic resistance. It is thus essential to identify new broomrape resistances acting at various stages of the interaction and combine them to improve resistance durability. In this study, 71 wild sunflowers and wild relatives accessions from 16 *Helianthus* species were screened in pots for their resistance to broomrape at the late emergence stage. From this initial screen, 18 accessions from 9 species showing resistance, were phenotyped at early stages of the interaction: the induction of broomrape seed germination by sunflower root exudates, the attachment to the host root and the development of tubercles in rhizotron assays. We showed that wild *Helianthus* accessions are an important source of resistance to the most virulent broomrape races, affecting various stages of the interaction: the inability to induce broomrape seed germination, the development of incompatible attachments or necrotic tubercles, and the arrest of emerged structure growth. Cytological studies of incompatible attachments showed that several cellular mechanisms were shared among resistant *Helianthus* species.

## Introduction

Sunflower, *Helianthus annuus*, is the fourth most important oil crop in the world after palm tree, soybean, and rapeseed (Rauf et al., 2017). Important genetic gain has been obtained over the last decades thanks to the discovery of the combination of cytoplasmic male sterility and restorer genes to produce hybrids (Terzic et al., 2020). Main genetic goals have focused on yield, oil content and quality, as well as tolerance to abiotic stresses such as drought or resistance to biotic stresses such as insects, oomycetes, fungi and more particularly broomrape (Skoric, 2016). The sunflower broomrape, *Orobanche cumana*, is a parasitic plant that is currently one of the main pests for sunflower crop, leading to major yield losses. Broomrape is present in the main countries where sunflower is cultivated with the remarkable exception of the American continent. Several control methods against broomrape have been developed (Fernández-Aparicio et al., 2020). Thus, parasitic weed management exploits herbicide strategies, such as Imidazolinone coupled with herbicide resistant sunflowers (Tan et al., 2005) and crop rotation including non-host trap crops inducing broomrape seed germination. However, the most efficient control of broomrape infestation relies on genetic resistance (Pérez-de-Luque et al., 2009).

The introduction of genetic resistances in sunflower breeding programs is linked to broomrape race evolution. Since the first breeding for broomrape genetic resistances to race A performed in Russia in the 1920s, vertical dominant resistant genes *HaOr1* to *HaOr7* allowing resistance to races A to F have been developed in a gene-for-gene approach (reviewed in Molinero-Ruiz et al., 2015; Cvejic et al., 2020). The intensification of sunflower cultivation and short crop rotations have speeded up the evolution of races. Nowadays, the most virulent races appeared in Eastern Europe and Spain, and were classified as G or H (Antonova et al., 2013; Kaya, 2014; Martín-Sanz et al., 2016). Genetic studies have revealed other resistances such as QTL to races F and G (Pérez-Vich et al., 2004; Louarn et al., 2016; Imerovski et al., 2019), or more recently single resistant genes to race G such as *Or_SII_* (Martin-Sanz et al., 2020) and *Or_Deb_2* (Fernandez-Aparicio et al., 2022). The only sunflower resistance gene identified so far is *HaOr7* encoding for a Leucine-Rich-Repeat Receptor-Like Kinase (Duriez et al., 2019).

*O. cumana* belongs to the Orobanchaceae, the largest family of parasitic plants with more than 2000 species (McNeal et al., 2013). *O. cumana* is an obligate root parasitic plant specific to sunflower in agricultural systems (Delavault, 2015). Its biological cycle can be divided into 4 main stages (Louarn et al., 2016). The 1^st^ stage starts with the seed germination. After exposure to adequate humidity and temperature conditions, the broomrape tiny seeds perceive germination stimulants (GS) produced by sunflower root exudates and germinate in the proximity of their host roots. Sunflower GS include a cocktail of molecules such as sesquiterpene lactones (for example dehydrocostus lactone-DCL and costunolide; Joël et al., 2011; Wu et al., 2022) and strigolactones (such as heliolactone, Ueno et al., 2014). During the 2^nd^ stage, once the seeds have germinated, the radicle develops and orientates itself toward the host root by chemotropism (Krupp et al., 2021). Following contact with the host root, the radicle apex develops papillae cells that attach to the host root. Then, the haustorium, a specific parasitic organ, penetrates into the host root, thanks to a combination of mechanical pressure and enzymatic excretion leading to host cell wall smoothing (Losner-Goshen et al., 1998; Brun et al., 2021). Haustorium intrusive cells reach the central cylinder of the host root, and vascular connections are established to highjack xylem and phloem saps to the benefit of the parasitic plant (Krupp et al., 2019). During the 3^rd^ stage, the attachment swells into a tubercle developing a bud. The bud develops into a single flowering stem, which emerges out of the soil, flowers, and produces thousands of tiny seeds during the last 4^th^ stage.

Most of the screenings for genetic resistances have been performed in fields or pots, at a late stage of broomrape emergence corresponding to the 4^th^ stage, with no information on the early stages of the interaction. However, in some studies, phenotyping was done in rhizotrons (small culture boxes) at early stages of resistance. Thus, it was shown that the *HaOr7* resistance gene (Duriez et al., 2019) and *Or_Deb2_* (Fernandez-Aparicio et al., 2022) led to resistance at the 2^nd^ stage leading to incompatible attachments for which vascular connections were not established. Tubercle necrosis was shown in the resistant line LR1 (Labrousse et al., 2001; Louarn et al., 2016). Moreover, the resistant gene *Or_SII_* affected the development of tubercles as well as the 4^th^ stage leading to emergence necrosis before flowering. In addition, a few studies led to a better understanding of the resistant mechanisms at the cellular and molecular levels. Incompatible attachments were associated with host defense responses at the tip of the haustorium, with an accumulation of phenolic compounds (Echevarria-Zomeño et al., 2006). An encapsulation layer around the haustorium (Dörr et al., 1994; Labrousse et al., 2001; Fernandez-Aparicio et al., 2022) and a gum-like substance deposition in host xylem vessels or the modifications of xylem cell walls preventing haustorium development and vascular connection, have been shown in some resistant accessions (Dörr et al., 1994; Labrousse et al., 2001). Incompatible attachments were also associated with callose deposition, suberization, and protein cross-linking at the interface between parasite and host cells (Echevarria-Zomeño et al., 2006). Finally, in the resistant line LR1, the induction of three genes linked to detoxification of reactive oxygen species suggested an oxidative burst at the basis of the haustorium (Letousey et al., 2007). These works revealed that various cellular mechanisms are involved in early post-haustorial resistances.

As monogenic resistance genes (vertical resistances) can easily be overcome by the appearance of new broomrape races, it is important to combine various resistant genes in pyramidal resistance breeding approach, and therefore to associate multiple resistance mechanisms affecting various stages of the interaction in a single variety (Seiler, 2019). Moreover, new resistance genes to the most virulent broomrape races G and H, are needed. Seiler and Jan (2014) reviewed the resistance genetic resources present in wild *Helianthus* species, including resistances to the virulent broomrape races G. Wild *Helianthus* species have been collected for more than 50 years in North America, and the genus *Helianthus* has a wealth of 53 species: 14 annual and 39 perennial, and 19 subspecies (Schilling, 2006; Stebbins et al., 2013).

Therefore, the present study was conducted to seek for resistances to broomrape in wild *Helianthus* species by screening a collection of accessions, at different stages of the interaction. The first objective of this study was to confirm the resistances to broomrape at the late emergence stage described in Seiler and Jan (2014), by screening a collection of 71 wild *Helianthus* accessions from 16 species in pots. The originality of our work was to perform in addition multiple phenotypings at various early stages of the interaction, using a subset of 18 resistant accessions from 9 species. Two broomrape races were used for the phenotyping screenings in pots and for the root exudate assays, that were chosen among 5 races, because of their contrasting perception of GS (strigolactone: GR24 or sesquiterpene lactones: DCL/costunolide), and one race was used for phenotyping in rhizotrons at the attachment and tubercle stages. We characterized the resistance mechanisms and identified four main groups of accessions.

## Materials and methods

### Plant materials

We used 36 accessions of wild *H. annuus* and 35 accessions of wild *Helianthus* other than *H. annuus* (1 to 3 accessions/taxon) from 15 species (19 taxa). Eight annual species (12 taxa): *H. anomalus, H. bolanderi, H. argophyllus, H. petiolaris* subsp.*petiolaris, H. praecox* subsp. *hirtus, H. praecox* subsp. *runyonii, H. praecox* subsp. *praecox, H. debilis* subsp. *tardiflorus, H. debilis* subsp. *debilis, H. debilis* subsp. *cucumerifolius, H. exilis, H. neglectus*, and 7 perennial species (7 taxa): *H. grosseserratus, H. tuberosus, H. divaricatus, H. nuttallii, H. pauciflorus* subsp. *subrhomboideus, H. strumosus* and *H. winteri*). The list of accessions and associated phenotyping assays are detailed in **Supplementary File 1**. The choice of wild *Helianthus* species relied on previous publications based on phenotyping studies using the most virulent races F and G (reviewed in Seiler and Jan, 2014), geographical diversity, PI (USA code) availability, and availability of seeds in the Biological Resource Centre of Sunflower in Toulouse (France; Terzic et al., 2020).

Five accessions were used as susceptible controls for the pot experiments in the first 2 assays (No. 1 to 2): NR5 (an inbred line carrying the gene *HaOr5* that provides resistance to race E; Tang et al., 2003); Hybrid 2 (Martín-Sanz et al., 2016), a commercial hybrid widely used in Southern Spain resistant to race F and susceptible to race G-GV (from Spain); P96, an inbred line resistant to races F and G-GV and susceptible to populations of race G from Eastern Europe (Martín-Sanz et al., 2016); LP2 (an inbred line carrying the gene *HaOr7* that confers resistance to race F and also incomplete resistance to populations of race G; Duriez et al., 2019), and Deb2 (an inbred line carrying the gene *HaOr_deb2_*, and resistant to race G; Fernandez-Aparicio et al., 2022). In the assay No. 3, B117 (a confectionery landrace susceptible to all broomrape populations) was used as susceptible control and LP2 as resistant. Two cultivated *H. annuus*, XRQ (Badouin et al., 2017) and 2603 from INRAE, were used as susceptible controls in the experiments in rhizotrons.

Five races of *O. cumana* from various countries were used and were named by the virulence level and the city or country of origin. The French race, E-BOU, (reference: LIPM-20734), representative of French races, classified between E and F, was harvested in Bourret (Tarn et Garonne, France) in 2017. Four other races of virulence G, reflecting the geographical diversity of the G races, were used: the race G-RO from Romania (harvested in Turcoaia (45.11584N/28.152629E) in 2018 (reference: LIPM-22957) kindly provided by Caussade-Semences, and multiplied in pots in 2019 (reference: LIPM-Oc00007); the race G-GV from Spain harvested in Villanueva del Rey, Seville Province in 2010 (Guadalquivir Valley; references LIPM-Oc00008 or IN230), the race G-RU from Russia harvested in Tatsinskiy, Rostov Province (reference: LIPM-Oc00006) and the race G-TK from Turkey collected in Çeşmekolu, Kirklareli Province, Turkey in 2000 (reference : LIPM-Oc00009). The differential resistant sunflower line for the races G was LP2.

### *O. cumana* races characterization

*O. cumana* seeds were prepared and conserved as in Huet et al. (2020). The seed lots were conserved in glass jars in a dry culture chamber at 25°C, and kept in the dark. To characterize *O. cumana* seed populations, the activity of three GS, a synthetic strigolactone (GR24; kindly provided by François-Didier Boyer; CNRS, ICSN, Gif/Yvette, France) and two sesquiterpene lactones (dehydrocostus lactone-DCL, D1627, LKT Laboratories Inc, St. Paul, USA; and costunolide, SML0417, Sigma-Aldrich, Inc., St. Louis, USA), was determined using the method described in Pouvreau et al. (2021) with slight modifications. Briefly, 200 mg of *O. cumana* seeds were sterilized and conditioned in 15 mL of medium (Hepes 1mM, PPM 0.1%, pH = 7.5) (PPM, Plant Preservative Mixture; Kalys, Bernin, France) for at least 7 days and no more than 6 weeks at 21°C in the dark. GS were resuspended in acetonitrile at 10 mM, then diluted with water at 100 µM (water/acetonitrile; v/v; 99/1). Three equimolar mixtures of GR24:DCL, GR24:costunolide and DCL:costunolide were also prepared at 100 µM. Dilutions from 10 µM to 1 pM were then prepared in water/acetonitrile (v/v; 99/1). For each compound and mixture, a range of concentrations from 1 µM to 0.1 pM (water/acetonitrile; 999/1) were applied to conditioned parasitic seeds. One µM of GR24 and 0.1% acetonitrile were used as positive and negative control, respectively. Three biological (germination assays) and three technical (dilutions) replicates were performed resulting in n=9. For each compound or mixture assayed, dose-response curves [GS activity = f(c); GS activity, Germination Stimulant activity relative to 1 µM GR24; c, concentration in M)], half maximal effective concentration (EC50), and maximum of GS activity were determined as previously described (Pouvreau et al., 2021). Germination percentage of each population was first determined with 1 µM GR24 in order to normalized all the subsequent results and avoid batch-to-batch variations related to seed maturation, age or seed viability.

### Phenotyping assay in 6 L pots at the emergence stage

Wild *Helianthus* seeds were surface sterilized, scarified and soaked in gibberellic acid (GA3) as in Ruso et al. (1996). The seeds were germinated in moistened filter paper and sown in small pots (7 x 7 x 7 cm) containing a mixture of sand and peat (1/1 v) together with 50 mg of *O. cumana* seeds. The plants were grown in a culture chamber for 20-25 days at 25 °C/20 °C (day/night) using a 16 h photoperiod, before their transfer to larger pots containing 6 L of soil mixture made of sand, silt and peat (2/1/1 v) and 8 g of NPK controlled release fertilizer Nutricote^®^ 15-9-10 (2MgO)+ME. The pots were placed in outdoor conditions. The plants were watered when required to avoid water stress. Broomrape resistance was evaluated by counting the number of emerged broomrape shoots for each sunflower plant at the end of sunflower flowering. In most cases, 6 plants were grown per accession and per race, but due to low germination ability of wild *Helianthus*, the number of phenotyped plants varied between accessions. Three independent assays were performed in the pot experiments in Cordoba (Spain). Two assays (No. 1 and No. 2) were performed during the spring and summer 2019 with the broomrape races E-BOU (2017) and G-RO (2018). The assay No. 3 was performed during the spring and summer 2021 with the four races G: G-RO (2019), G-GV, G-RU and G-TK, using 5 to 8 pots/race/accession.

### Preparation of *Helianthus* root exudate and phenotyping of *Helianthus* root exudate activity on broomrape seed germination

Wild *Helianthus* seeds were surface sterilized, scarified and soaked in GA3 as in Ruso et al. (1996). The seeds of *Helianthus annuus* accession XRQ were prepared without the scarification procedure. Six seedlings of each accession were disposed individually into drained plastic pots [700 ml, 9 × 11 × 12 cm (W×L×H), Nicoplast] nested in undrained pots and filled with glass beads (Ø 3 mm) covered by a 1 cm layer of vermiculite. The pot systems were deposited in a growth chamber at 21°C with a 16 h light/8 h dark photoperiod. Plantlets were first watered with 200 ml of half-strength Long Ashton solution (Hewitt, 1966) supplemented with 370 µM of phosphate and the volume was renewed every two days. Culture medium (fresh root exudates) from each drained pot was recovered once a week during four weeks (at 3 weeks to 6 weeks after sowing) and assayed directly to evaluate its capacity to stimulate the seed germination of *O. cumana* populations.

GS activity of fresh *Helianthus* root exudates was determined weekly on seeds of *O. cumana* using the method described in Pouvreau et al. (2021) with slight modifications. Briefly, 200 mg of *O. cumana* seeds were sterilized and conditioned in 15 mL of medium (Hepes 1mM, PPM 0.1%, pH = 7.5) for at least 7 days and no more than 6 weeks at 21°C in the dark. Conditioned seeds of parasitic plants were distributed in 96 well plates (50 µL/well) and 50 µL of fresh sunflower exudate filtered through a 0.22 µm PES membrane filter unit were added per well. Fifty µL of sterile water or GR24 plus DCL at final concentrations of 1µM each were added for negative and positive control, respectively. Plates were incubated 4 days in the dark at 21°C and 10 µL of 5 g/L MTT (3-[4,5-dimethylthiazol-2-yl]-2,5-diphenyltetrazolium Bromide, M2003, Sigma-Aldrich, Inc., St. Louis, USA) were then added per well. The yellow MTT is reduced to purple formazan crystals by mitochondrial enzymatic activities, indicating a metabolic recovery. This staining therefore facilitates the counting of germinated seeds. Thus, after 24 h at 21°C, the percentage of germination was determined by counting stained germinated seeds using a zoom stereo microscope. Each germination assay was normalized against positive and negative controls prepared each week and from the same sterilized seed lot. The plants were analyzed individually to avoid the mixing of different exudates in the case of segregating resistance. Concretely, one experiment consisted in 4 accessions, 3 wild type and the susceptible control line XRQ. For each accession 6 plants (6 pots with 1 plant) were grown independently during 6 weeks. Every week, the exudates from each plant were collected and independently assayed for GS activity.

### Phenotyping in rhizotrons at the attachment and tubercle stages

Plants were grown in rhizotrons as in Le Ru et al. (2021), with some modifications adapted to the slow growth of wild *Helianthus* species. A timeline of the experiment in rhizotron is presented in **Supplementary File 2**. Wild *Helianthus* seeds were sterilized according to Ruso et al. (1996). Wild *Helianthus* seeds were first surface sterilized in 4.8% sodium hypochlorite with 0.05% Triton X-100 as surfactant for 15 min and rinsed three times with sterile water. The seeds were then scarified by cutting 1/3 off the blunt end of the seed (cotyledon side) and soaked in a 100 mg/L solution of GA3 (Duchefa G-0907) for 4 hours. Then the seeds were placed in Petri dishes on a filter paper soaked with sterile deionized water, and incubated in the dark at 24°C for 3 days. After 3 days, the hulls were removed from the embryos and the embryos (germinated or not germinated) were sown in a mixture of 1/3 (v/v) sand/soil for 2 weeks at 22°C, 60% humidity, 16 h light, 118 µE/m²/s. Following these 2 weeks of culture, the germinated seedlings were transferred into the rhizotrons: 12 x 12 cm home-maid Plexiglas boxes containing water-soaked autoclaved rockwool (Grodan Expert, Grodan (Rockwool B. V) P.O. Box 1160, 6040 KD Roermond, The Netherlands) and a sterile glass fiber paper (Dutscher, reference 036294B) for 6 days, 1 plant/rhizotron. On the same day, the cultivated *H. annuus* control seeds (XRQ or 2603) were sterilized with 4.8% sodium hypochlorite for 10 min, rinsed three times with sterile water and sown in a mixture of 1/1 (v/v) sand/vermiculite at 22°C, 60% humidity, 16 h light 118 µE/m²/s for 6 days. On the same day, *O. cumana* seeds were sterilized for 5 min with 3.2% sodium hypochlorite and 0.01% Triton X-100, rinsed three times with sterile water using a 40 µm sterile cell strainer, and subsequently conditioned in water (3.3 mg/ml) for 6 days at 22°C in the dark in a 50 ml sterile tube. Six days later, the wild *Helianthus* plantlets in rhizotrons were inoculated with the water-conditioned *O. cumana* seeds (10 mg/rhizotron), and half of the rhizotrons were treated with a mixture of rac-GR24 (reference CX 23880; Chiralix, Nijmegen, the Netherlands) and Dihydrocostus Lactone (DCL, reference 42575, Sigma) to promote *O. cumana* seed germination (5 ml of 10^- 7^ M GR24 + DCL/rhizotron). Half of the rhizotrons did not receive any treatment. On the day of the inoculation, cultivated control plantlets were transferred to rhizotrons and inoculated as well (10 mg/rhizotron) without any treatment. The rhizotrons were placed vertically in a growth chamber at 22°C with 60% humidity (16 h day, 110 µE/m²/s) and watered 3 times per week with a half-strength Long Ashton nutrient solution (Hewitt 1966) supplemented with 370 µM phosphate. The rockwool dried between two watering periods.

Phenotyping was performed at 14, 21 and 28 days after inoculation (dai). Germination of *O. cumana* seeds was evaluated at 7 and 14 dai. Compatible and incompatible attachments were counted at 14 dai using a binocular (Leica S6D). At 21 and 28 dai, pictures of the whole root system were taken using a Raspberry/picamera as described in Le Ru et al. (2021) and the final number of tubercles and necrotic tubercles were counted using ImageJ software (version 1.52a; Schneider et al., 2012) and the magic wand tool. In addition, pictures were taken using a stereomicroscope (Axiozoom V16; Zeiss). Rhizotrons containing plants that were not well developed or that were different in their aerial phenotypes within the same accession were discarded from phenotyping. This was the case for the accession 584 *H. bolanderi*, for which 2 bigger plants were discarded (among 14 plants).

In each independent rhizotron experiment, 3 to 4 wild *Helianthus* accessions and a cultivated susceptible control (XRQ or 2603) were phenotyped, with 10 rhizotrons/accession (5 non-treated and 5 treated) when possible. Due to low germination ability, the number of rhizotrons varied among accessions and between experiments. The final numbers of rhizotrons [non-treated (NT) and treated (T)] and the number of independent experiments for each accession are listed in **Supplementary File 3**. At least 2 independent experiments were performed for each accession. Raw data are given in **Supplementary File 4**. Data were Not Available (NA) when the plant was dead, or following sampling of attachments for cytological studies. Data included the wild *H. annuus* #654 (susceptible in pots) and both susceptible cultivated control lines (XRQ and 2603, untreated rhizotrons only).

### Cytological studies

At 14 dai, 10 root segments with attachments (compatible or/and incompatible depending on the accession) were sampled for cytological studies from one rhizotron per accession. In the case of wild *H. annuus* accessions or of the cultivated control, samples were taken from a non-treated rhizotron as the root exudates of these accessions were inducing broomrape seed germination. When the accessions did not induce broomrape seed germination, attachments were only obtained from GS-treated rhizotrons. Two types of samples were prepared: cleared whole root segments and thin sections. Whole root segments were fixed in ethanol-acetic acid 3/1 (v/v) for 10 min under vacuum, cleared in chloral hydrate 5 g/ml and observed with an Axioplan 2 microscope (Zeiss). For thin sections, samples were fixed in FAA (36% formaldehyde, 5% acetic acid, and 50% ethanol), for 5 min under vacuum and rinsed in 70% ethanol. Sections were embedded in Technovit 7100 resin (Heraeus Kulzer, Wehrheim, Germany), according to the manufacturer’s recommendations. Thin sections (10 µm) were made using a microtome (2040 Reichert Jung), stained in 0.2% toluidine blue for 3 min, mounted in DePeX mounting medium (BDH Laboratories, Poole, England), and scanned using a Nanozoomer (NDP, Hamamatsu).

### Statistical analysis

Phenotyping data of *O. cumana* seed germination in response to GS or to *Helianthus* root exudates were analyzed as previously described (Pouvreau et al., 2021) using SigmaPlot 10.0 and SigmanStat 3.5.

Phenotyping data in rhizotrons were subjected to statistical analysis using R (R Core Team, 2021). Comparisons of accessions were characterized by the different random variables chosen: treatment effect, number of attachments, number of tubercles at 21 and 28 dai, number of tubercles, attachment efficiency at 21 and 28 dai: number of tubercles/number of attachments, and the percentage of necrotic tubercles at 28 dai. The same statistical approach was carried out for each variable. Normality, independence, and homogeneity of variances were systematically verified by diagnostic diagrams and by Shapiro test (Normality test, a=0.05) and Bartlett test (homogeneity of variances test). If the assumptions were verified, an ANOVA analysis was performed. If one of the hypotheses was not verified, the non-parametric test of Kruskal-Wallis was realized.

## Results

We screened a large collection of wild *Helianthus* species for resistance to *O. cumana.* We selected 36 wild *H. annuus* accessions for which introgressed line populations had been developed in previous projects and 35 wild *Helianthus* accessions from species other than *H. annuus*, including 21 accessions from 8 annual species and 14 accessions from 7 perennial species (**Supplementary File 1**). First, we characterized the perception of 5 sunflower broomrape races to GS which led to the selection of 2 races for the initial screening in pots at the emergence stage. Then, we further phenotyped 18 accessions resistant or segregating for the resistance at early stages of the interaction: (*i*) using a germination induction assays by root exudates and (*ii*) and phenotyping in rhizotrons at the attachment and tubercle stages.

### Contrasted perception of germinating stimulants among broomrape races

**Figure 1:**
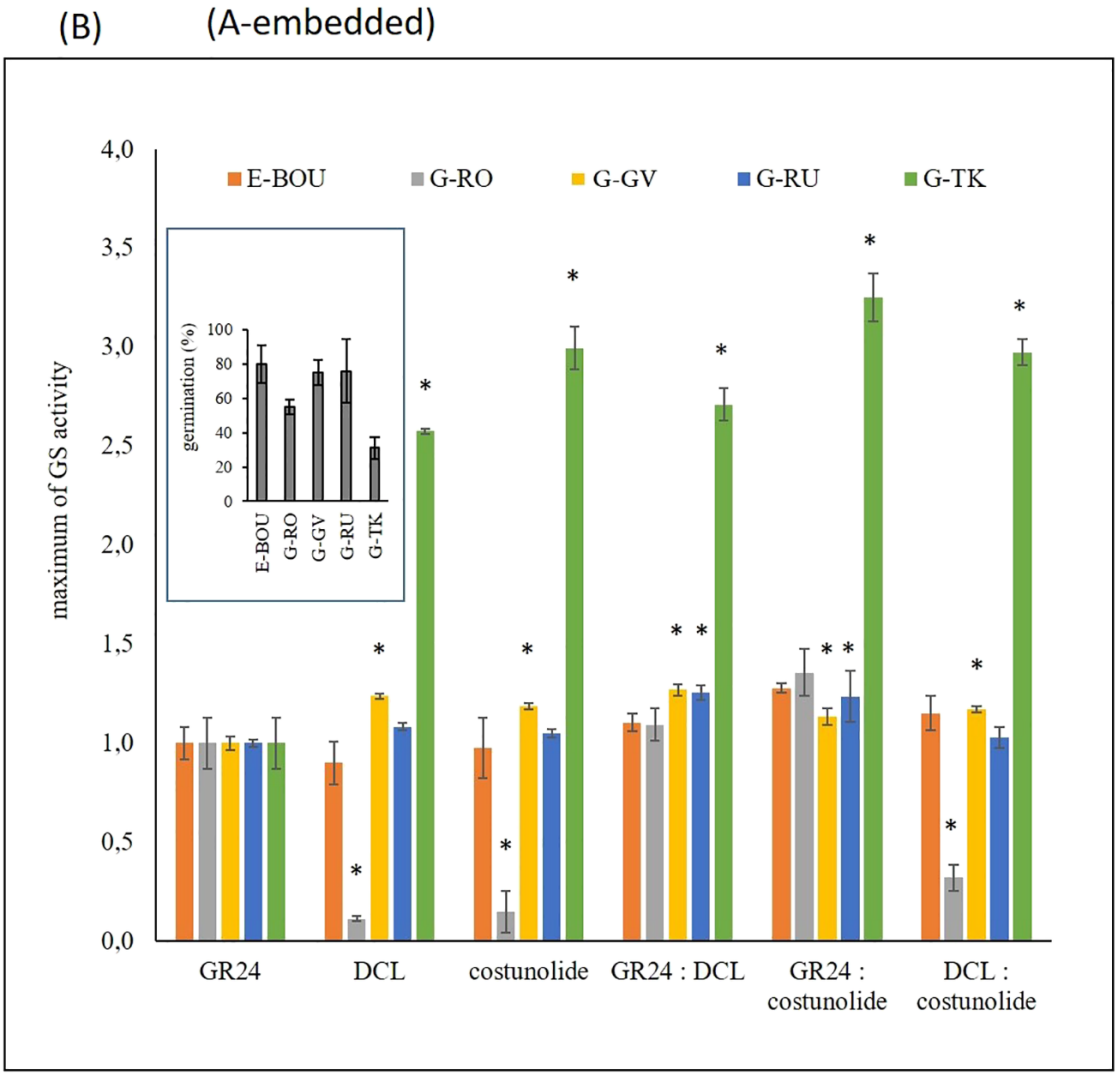
Seed germination of five *O. cumana* populations in response to various germination stimulants **(A)**. Percentage of seed germination of five *O. cumana* populations upon treatment with 1 µM GR24 (bar: ± SE). These results were used to normalize the germination stimulant activity in order to generate the dose-response curves (**Supplementary File 5**). **(B)** Maximum of germination stimulant activity of GR24, DCL, costunolide and equimolar mixtures of these compounds on the seeds of the five *O. cumana* populations (bar: ± SE) were determined from the dose-response curves (**Supplementary File 6**). Holm-Sidak method was used *versus* the control group (asterisks indicate significant differences between GR24 and compounds or mixtures (p < 0.05) for each race).

Five broomrape populations (1 race E: E-BOU; and 4 races G: G-RO, G-GV, G-RU and G-TK) were phenotyped for the capacity of their seeds to germinate when different GS were applied: GR24, a synthetic strigolactone used in most studies for parasitic seed germination induction (Mangnus et al., 1992), and two sesquiterpene lactones identified in sunflower (dehydrocostus lactone-DCL and costunolide; Raupp and Spring, 2013). The germination percentage of each population, determined initially with 1 µM GR24, varied from 31 % to 80% (**Figure 1A**). Seed batch quality and/or sensitivity to GR24 could explain the variability of germination observed among the various races. With these results, dose-response curves were modelled by testing GS separately (GR24, DCL, and costunolide) or in equimolar mixtures of two compounds at concentrations ranging from 1 µM to 0.1 pM (**Supplementary File 5**). Maximum GS activity relative to 1 µM GR24 (**Figure 1B**) and half maximal effective concentration (**Supplementary File 6**) were then determined for each GS and *O. cumana* population. Three populations, E-BOU, G-GV, and G-RU, responded to the 3 compounds (GR24 or DCL or costunolide) in the same way by reaching the maximum activity. By contrast, for the G-TK population, both sesquiterpene lactones, DCL, and costunolide, induced germination 2.6 to 3 times higher than GR24 (80.6 and 93% of seed germination, respectively). Conversely, the G-RO population responded less to DCL and costunolide, since only 6 and 8% of maximum seed germination were observed, respectively. Co-stimulations (GR24:DCL, GR24:costunolide and costunolide:DCL) did not induce synergy on sensitivity. Population sensitivities to GS were variable, with the G- TK population being the least responsive to GR24 (EC50, 2.2 ± 0.8 10^-9^M). Conversely, the response to sesquiterpene lactones was low for the G-RO population and the sensitivity low for E-BOU (**Supplementary File 6**).

In summary, a diversity of responses to GS was highlighted among the populations of *O. cumana*: three populations (E-BOU, G-GV, and G-RU) were sensitive to strigolactone and sesquiterpene lactones, a population (G-RO) responded mainly to strigolactones, and a population (G-TK) showed an optimal response to sesquiterpene lactones. Because of these contrasting responses and the constraints linked to the availability of seeds for G-TK, the race G-RO was used as an indicator of the exudation of strigolactones and E-BOU of the presence of the 2 types of compounds, strigolactones and sesquiterpene lactones. Due to the variability of responses to GS, co-stimulation with GR24 and DCL was used as a reference for the exudation monitoring and experiments in rhizotrons.

### Wild *Helianthus* species: A large genetic resource for resistance to *O. cumana*

**Figure 2:**
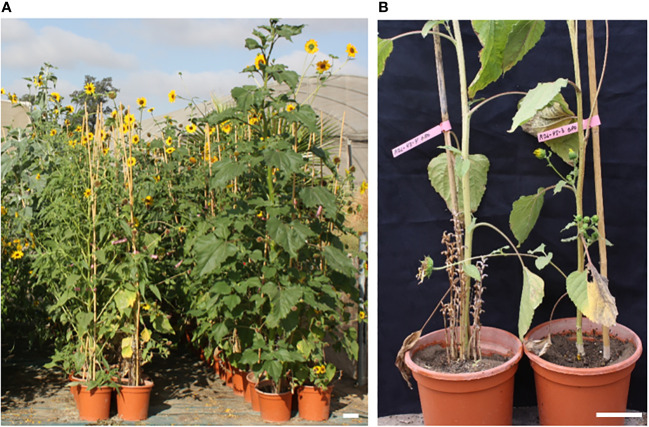
Phenotyping wild *Helianthus* species in 6 L pots for their resistance to *O. cumana.* **(A)** Global view of a phenotyping assay in 6 L pots of wild *Helianthus* species. Screening was performed by counting the number of emergences/pot at the flowering stage of wild *Helianthus.* **(B)** Segregating resistance of the *H. annuus* accession #774: on the left, susceptible plant with more than 10 emergences and on the right, resistant plant with no emergence. Bar = 10 cm.

The wild *Helianthus* accessions were screened at the emergence stage in 6 L pots (**Figure 2**) by counting the number of broomrape emergences in each pot at the flowering stage of *Helianthus* plants. The screening was done using the broomrape races E-BOU and G-RO showing contrasted GS perception as stated above.

Among the 36 wild *H. annuus* accessions phenotyped, 4 accessions segregated for the resistance with at least 2/6 pots with no emergence (**Table 1**): #833, #774, #2008 and #2016. The accessions #833 and #2008 showed the highest level of resistance to both races. Moreover, the accessions #774 and #826 showed a late resistant phenotype leading to the arrest and necrosis of the growth of broomrape apex, similar to the phenotype described for the resistant gene *Or_SII_* (Martin-Sanz et al., 2020). For the accession #774, 6/6 and 4/6 plants showed late resistance to race E-BOU and G-RO, respectively. For the accession #826, the late resistance phenotype was only observed with the race E-BOU, and not with the race G-RO. In conclusion, few resistances could be observed among wild *H. annuus* species. By contrast, the large majority of the wild *Helianthus* species other than *H. annuus* were resistant to both races, with the remarkable exceptions of *H. argophyllus* which was susceptible and *H. winteri* which segregated for the resistance (**Table 2**). No major difference could be observed between both races, suggesting non-race-specific resistances among wild *Helianthus* species. From this initial screening, 18 accessions from 9 species (including *H. annuus*) resistant or segregating for the resistance were selected for further phenotyping assays.

**Table 1:** Results for the phenotyping of wild *Helianthus annuus* species in 6 L pots to *O. cumana* races E-BOU and G-RO.

ID French Seed Bank	PI (ID USA)	* *species	No. assay	E-BOU						G-RO					
				Plant-1	Plant-2	Plant-3	Plant-4	Plant-5	Plant-6	Plant-1	Plant-2	Plant-3	Plant-4	Plant-5	Plant-6
351		*H. annuus*	1	S	4	S	S	S		S	S	1	1	S	S
358		*H. annuus*	1	S	S	S	S	S	S	S	S	S	S	S	S
421		*H. annuus*	1	S	S	S	5			S	S	S	S	S	7
649		*H. annuus*	1	S	S	S	S	S	S	S	S	S	S	S	0
650		*H. annuus*	1	S	S	2	S	S	S	S	S	S	S	S	S
654		*H. annuus*	1	S	S	1	S	S	S	S	S	6	S	S	5
661		*H. annuus*	1	S	S	S	S	S	S	S	S	S	S	S	S
662		*H. annuus*	1	S	S	S	S	S		S	S	S	S	S	
775		*H. annuus*	1	S	S	S	S	S	8	S	S	S	S	S	S
829		*H. annuus*	1	S	S	S	S	S	S	S	S	S	S	S	S
831		*H. annuus*	1	S	S	S	S	S		S	S	S	S	S	
**833**		***H. annuus lenticularis***	1	3	5	0	S	0	0	1	0	2	1	0	S
928		*H. annuus*	1	S	2	S	S	S	S	S	S	S	S	S	S
955		*H. annuus*	1	3	S	S	S	S		1	S	S	S	S	
963		*H. annuus*	1	S	S	S	S	S		S	S	S	S	S	S
980		*H. annuus*	1	S	S	S	S	S	S	S	S	S	S	S	S
999		*H. annuus*	1	S	S	S	S	4	S	S	S	S	S	S	S
1016		*H. annuus*	1	S	S	S	S	S	S	S	S	S	S	S	S
774*		***H. annuus***	1	0	10	2	7	0	2	7	4	5	S	2	S
826**		***H. annuus***	1	8	S	5	S	7	2	0	S	S	10	1	S
Idaho		*H. annuus*	1	S	5	S	S	S	S	S	S	S	S	1	S
2000	PI 413021	*H. annuus*	2	S	S	S	S			S	S	S	S	S	S
2001	PI 413095	*H. annuus*	2	S	S	S	S	S	S	2	S	S	S	S	3
2002	PI 413131	*H. annuus*	2	S	S	S	8	S	S	S	S	S	S	S	S
2003	PI 435368	*H. annuus*	2	S	6	S	S			10	S	S	4	0	
2004	PI 435457	*H. annuus*	2	3	S	S	S	S		S	S	S	S	S	
2005	PI 435531	*H. annuus*	2	S	S	S				S	S	S			
2007	PI 435850	*H. annuus*	2	S	S	S	S	S	S	S	S	S	S	S	S
**2008**	**PI 468571**	***H. annuus***	2	0	S	0	0	0	S	0	0	S	0		
2010	PI 586809	*H. annuus*	2	S	S	S	S	S		S	S	S	S	S	S
2011	PI 586819	*H. annuus*	2	S	S	7				S	S	S	S		
2012	PI 586879	*H. annuus*	2	S	S	S	S			S	S	S	S	S	
2013	PI 592312	*H. annuus*	2	S	S	S	6	S		S	4	S	S		
2014	PI 613752	*H. annuus*	2	S	S	S	S	5	S	S	3	1	S	S	0
2015	PI 613783	*H. annuus*	2	S	S	S	S	S	S	S	S	S	S	S	
2016/2017	PI 649814	*H. annuus*	2	S	0	3	S	S	S	1	6	S	0	0	
NR5		control Or5	1	4	0	1	2	2	0	S	S	S	S	S	S
NR5		control Or5	2	1	5	1	1	3	3	S	S	S	S	S	S
Hybrid2			1	0	1	3	1	4	2	S	S	S	S	S	S
Hybrid2			2	0	0	3	0	0		5	2	2	1	2	
P96		control to race F	1	0	0	0	0	0	0	4	1	6	3	3	0
P96		control to race F	2	0	0	0	0	0	0	0	0	0	0	0	0
LP2		control Or7	1	0	0	0	1	0	0	3	7	4	4	2	8
LP2		control Or7	2	0	0	0	0	0	0	0	0	0	0	0	0
Deb2		control deb2	1	0	0	0	0	0		0	0	0	0	0	
Deb2		control deb2	2	0	0	0	0	0	0	0	0	0	0	0	0

Two assays (No. 1 and 2) were performed during the spring/summer 2019. In each pot, the number of broomrape emergences was counted at the time of sunflower flowering. S: susceptibleaccession (≥ 15 emergences). In green: pots with no emergence. In bold: accessions chosen for phenotyping of exudate activity and in rhizotrons.

**Table 2:** Results for the phenotyping of wild *Helianthus* species (other than *H. annuus*) in 6 L pots to *O. cumana* races E-BOU and G-RO.

ID French Seed Bank	PI (ID USA)	* *species	* *No. assay	E-BOU						G-RO					
				Plant-1	Plant-2	Plant-3	Plant-4	Plant-5	Plant-6	Plant-1	Plant-2	Plant-3	Plant-4	Plant-5	Plant-6
525	PI 468638	*H. anomalus*	1							0					
2100	PI 468638	*H. anomalus*	2	0						0					
861		*H. argophyllus*	1	S	S	S	S	S		S	S	S	S	3	S
2200	PI 435629	*H. argophyllus*	2	S	S	S	S	S		S	5	5	3	4	
255		*H. bolanderi*	1	0	0										
**584**		***H. bolanderi***	1	0	0	0	0	0	0	0	0	0	0	0	0
**588**		***H. bolanderi***	1	1	0	0				0	0	0			
2301	PI 435641	*H. bolanderi*	2	0	0	0	0	0	0	0	0	0	0	0	0
2400	PI 653609	*H. debilis cucumerifolius*	2	0	0	0	0	0	0	0	0	0	1		
835	PI 435671	*H.debilis debilis*	1	0	0	0	0	0		S	0	2	0	0	0
**786**	**PI 468691**	***H. debilis tardiflorus***	1	0	0	0	0	0		0	0	0	0	0	0
**837**	**PI 468689**	***H. debilis tardiflorus***	1	6	0					6	2				
232	PI 435675	*H. divaricatus*	1	0						0	0				
**783**	**PI 435675**	***H. divaricatus***	1	0							0	0			
2600	PI 649895	*H. exilis*	2	1	0	1				0	0	0	0		
**2601**	**PI 664629**	***H. exilis***	2	0	0	0	0	0		0	0	0	0	0	0
**290**		***H. grosseserratus***	1	0	0	0	0	0		2	0	0			
1014		*H. grosseserratus*	1	0	0	0	0	0	0	0	0	0	0	0	0
1036		*H. grosseserratus*	1	0	0	0	0			0	0	0	0	0	
2700	PI 435768	*H. neglectus*	2	0						0	0				
2701	PI 597916	*H. neglectus*	2							0	0	0			
239	PI 435779	*H. nuttallii*	1	0	0					0	0				
1217	PI 531047	*H. nuttallii*	1	0	0	0	0	0		0	0	0	0	0	
926		*H. pauciflorus subrhomboideus*	1	0	0	0	0	0	0	0	0	0	0	0	0
969		*H. pauciflorus subrhomboideus*	1	0	0	0	0	0	0	0	0	0	0	0	0
1031		*H. pauciflorus subrhomboideus*	1	0	0	0	0			0	0	0	0		
**736**	**PI 468823**	***H. petiolaris petiolaris***	1	0	0	0	0			0	0	0	0		
**761**		***H. petiolaris petiolaris***	1	0		0	0	0	0	0	0	0	0	0	0
198		*H. praecox hirtus*	1	0	2	0	3	0	1	1	S	0	0	0	0
**677**	**PI 468850**	***H. praecox hirtus***	1	0	0	0	0			0	0	0	0	0	0
**3000**	**PI 468851**	***H. praecox praecox***	2	0	0	0				0	0	0	0	0	
**679**	**PI 468860**	***H. praecox runyonii***	1	0	0	0	0	0	0	1	0	0	0	2	0
1225		*H. strumosus*	1	0	0	0	0	0	0	0	0	0	0	0	0
**325**		***H. tuberosus***	1	0	0	0	0	0	0	0	0	0	0	0	0
**1013**		***H. tuberosus***	1	0	0	0	0			0	0	0	2	0	
3100	PI 673292	*H. winteri*	2	S	3	S	0	S	1	1	S	4	S	S	0
NR5		control Or5	1	4	0	1	2	2	0	S	S	S	S	S	S
NR5		control Or5	2	1	5	1	1	3	3	S	S	S	S	S	S
Hybrid 2			1	0	1	3	1	4	2	S	S	S	S	S	S
Hybrid 2			2	0	0	3	0	0		5	2	2	1	2	
P96		control to race F	1	0	0	0	0	0	0	4	1	6	3	3	0
P96		control to race F	2	0	0	0	0	0	0	0	0	0	0	0	0
LP2		control Or7	1	0	0	0	1	0	0	3	7	4	4	2	8
LP2		control Or7	2	0	0	0	0	0	0	0	0	0	0	0	0
Deb2		control deb2	1	0	0	0	0	0		0	0	0	0	0	
Deb2		control deb2	2	0	0	0	0	0	0	0	0	0	0	0	0

Two assays (No. 1 and 2) were performed during the spring/summer 2019. In each pot, the number of broomrape emergences was counted at the time of sunflower flowering. S: susceptibleaccession (≤ 15 emergences). In green: pots with no emergence. In bold: accessions chosen for phenotyping of exudates and in rhizotrons.

### Inactive root exudates in wild *Helianthus* species

**Figure 3:**
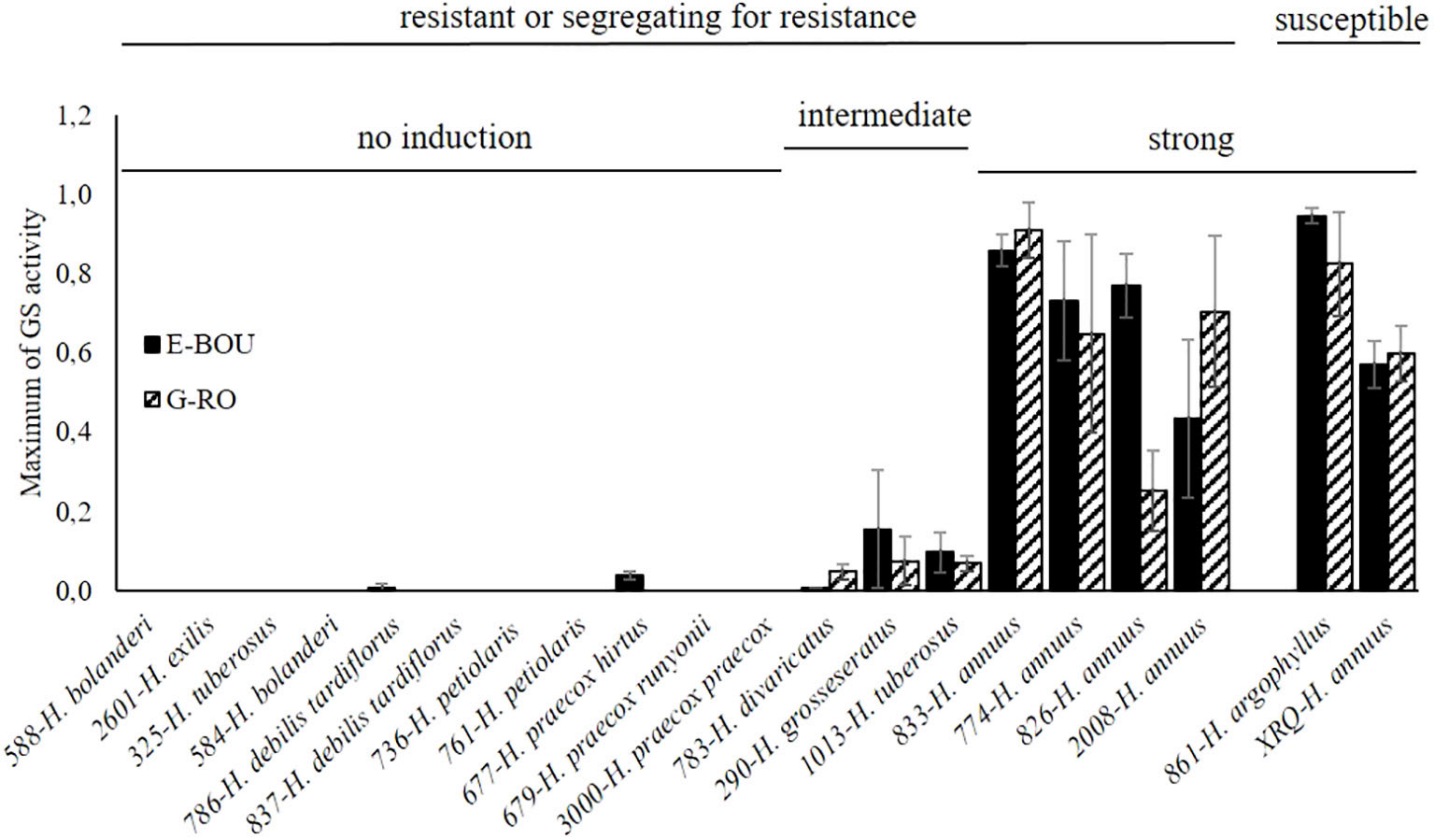
Root exudate activity of various *Helianthus* species on seed germination of two *O. cumana* populations. Maximum of germination stimulant activity of root exudates of 18 *Helianthus* accessions, 1 susceptible *H. argophyllus* and the susceptible control XRQ, on the seeds of the two *O. cumana* populations, E-BOU and G-RO (bar: ± SE), were determined from the time-response curves presented in the **Supplementary File 7**. The average activity of root exudates of the most active week was used for the **Figure 3**.

Because the earliest step involved in the susceptibility or the resistance of a plant host to a root parasitic plant is its ability to induce or not the seed germination of the parasite, we decided to evaluate this feature in a subset of 18 accessions resistant or segregating for resistance in pots. To this end, root exudates were produced under hydroponic conditions and tested as GS on seeds of both races of *O. cumana*, E-BOU and G-RO. In addition, we used a susceptible wild *H. argophyllus* (#861; evaluated in pots, **Table 2**) and the cultivated *H. annuus* XRQ as a susceptible control. In order to determine a maximum of GS activity on the two *O. cumana* populations comparable between the different accessions, exudate activities were normalized with the results obtained with 1 µM GR24:DCL (**Supplementary File 5**). As shown in **Figure 3** and **Supplementary File 7**, the 18 accessions fell into 3 groups: 4 accessions (*H. annuus*) with root exudates that strongly stimulated seed germination, 3 accessions (*#*783 *H. divaricatus*, *#*290 *H. grosseserratus*, and *#*1013 *H. tuberosus*) with low stimulatory activity, and 11 accessions belonging to wild *Helianthus* species other than *annuus* without activity. As expected the roots of the two susceptible accessions (*H. argophyllus #*861 and the cultivated control *H. annuus* XRQ) strongly stimulated the seed germination of both races. It should be noted that all *H. annuus* genotypes belonged to the group with strong activity. Moreover, in most cases, there was no significant race specificity between E-BOU and G-RO, except for a few accessions (*#*677 *H. praecox*, *#*783 *H. divaricatus* and *#*826 *H. annus*). It is interesting to highlight the divergent pattern of two *H. tuberosus* accessions: #1013 induced moderately seed germination of both races while #325 exhibited no activity.

### Early resistances at the attachment and tubercle stages in wild *Helianthus* species

**Figure 4:**
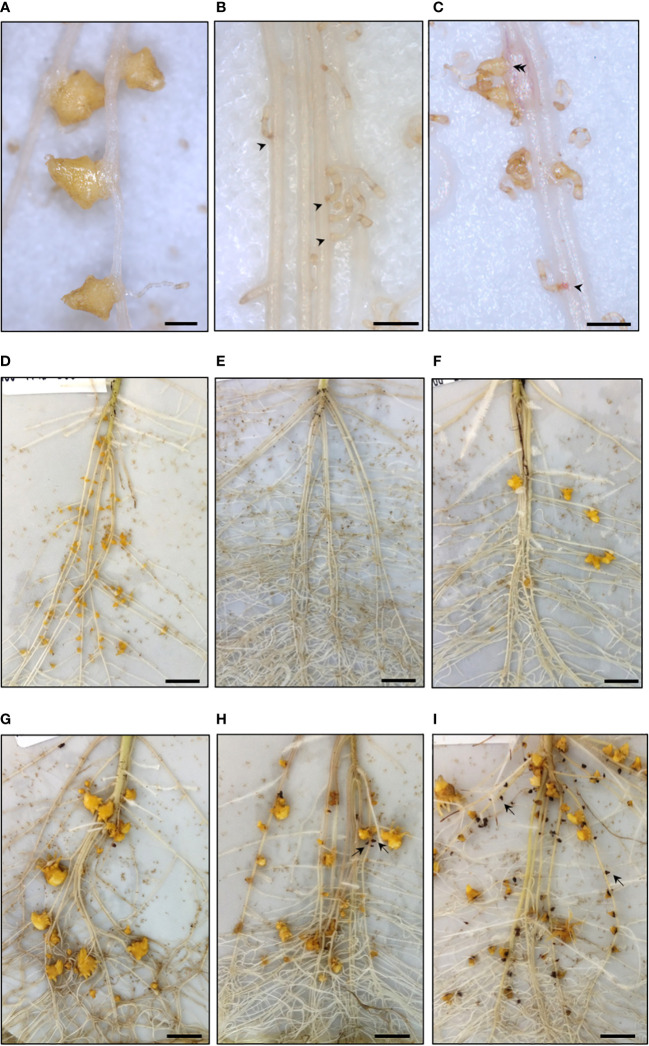
Early resistance stages of wild *Helianthus* species phenotyped in rhizotron using the broomrape race E-BOU. **(A–C)** Attachments at 14 dai. **(A)** Compatible attachments, accession #826 *H*. *annuus* (rhizotron non-treated). **(B)** Incompatible attachments, accession #783 *H*. *divaricatus* (arrow heads). **(C)** Mix of incompatible (arrowhead) and compatible attachments (double arrowhead), accession #761 *H*. *petiolaris*. **(D–F)**. Tubercles at 21 dai. **(D)** High number of tubercles, accession #774 *H*. *annuus.* **(E)** Absence of tubercles, accession #2601 *H*. *exilis*. **(F)** Low number of tubercles, accession #736 *H*. *petiolaris*. **(G–I)** Necrotic tubercles at 28 dai (examples indicated by arrows). **(G)** Absence of necrotic tubercles, accession #774 *H*. *annuus* (rhizotron non-treated). **(H)** Low percentage of necrotic tubercles, accession #677 *H*. *praecox*. **(I)** High percentage of necrotic tubercles, accession #786 *H*. *debilis*. A–C. Bar = 1 mm. D–I. Bar = 1 cm. All pictures were taken from rhizotrons treated with GR24+ DCL, except for **Figures 4A and G** taken from non-treated rhizotrons.

We performed experiments in rhizotrons allowing us to phenotype precisely the attachment and tubercle stages. The 18 accessions tested above, one susceptible wild *H. annuus* (#654; evaluated in pots, **Table 1**) and the cultivated sunflower susceptible controls (XRQ or 2603) were phenotyped, using the race E-BOU, representative of the French races. Since most of the wild accessions did not induce germination, half of the rhizotrons were treated the day of inoculation with GS (GR24 + DCL) to induce germination of broomrape seeds and identify potential resistances affecting stages following germination. The various phenotyping stages observed in rhizotrons are illustrated in **Figure 4**. The number of attachments (14 days after inoculation; dai), the number of tubercles (21 dai) and the percentage of necrotic tubercles (28 dai) in rhizotrons with (Treated) or without GS treatment (Non-Treated) are shown in **Tables 3** and **4** and the raw data are detailed Suppl File 4.

**Table 3:** Quantitative data of the phenotyping in rhizotrons of 18 wild *Helianthus* accessions from 9 species at the attachment and tubercle stages, using the broomrape race E-BOU.

Accession	Species	Class	Non-treated						Treated(GR24 + DCL)						
			Attachments -14dai			Tubercles-21 dai			Attachments -14dai			Tubercles-21dai			
			mean	SEM	*	mean	SEM	*	mean	SEM	*	mean	SEM	*
588	*H. bolanderi*	I	0		(8)	0		(8)	15,5	+/- 2,5	(2)	0		(2)
2601	*H. exilis*	I	1	+/- 0,4	(8)	0		(8)	60,7	+/- 8,7	(12)	0		(11)
325	*H. tuberosus*	I	0		(8)	0		(8)	23,7	+/- 3,8	(7)	0		(6)
584	*H. bolanderi*	II	0,4	+/- 0,2	(5)	0		(5)	21,4	+/- 5	(7)	1,3	+/- 0,5	(6)
786	*H. debilis tardiflorus*	II	0		(5)	0,8	+/- 0,4	(5)	49,6	+/- 10,2	(8)	35,9	+/- 11,7	(8)
837	*H. debilis tardiflorus*	II	0,3	+/- 0,2	(12)	0,6	+/- 0,2	(12)	38,6	+/- 5,6	(9)	30,8	+/- 2,1	(8)
736	*H. petiolaris*	II	0		(10)	0		(10)	19,4	+/- 5,3	(7)	3,3	+/- 1,4	(7)
761	*H. petiolaris*	II	0		(14)	0		(14)	21,9	+/- 2	(11)	9,8	+/- 2,2	(10)
677	*H. praecox hirtus*	II	0		(8)	0,8	+/- 0,5	(8)	19,2	+/- 2,9	(9)	7,2	+/- 2,6	(9)
679	*H.praecox runyonii*	II	1,5	+/- 0,6	(12)	1,4	+/- 0,6	(12)	36,3	+/- 6,3	(10)	28,6	+/- 7	(10)
3000	*H. praecox praecox*	II	0		(7)	0,2	+/- 0,1	(7)	28,7	+/- 5,3	(7)	6,9	+/- 1,7	(7)
833	*H. annuus*	III	25,2	+/- 6,5	(5)	0		(5)	56,6	+/- 6,9	(8)	0,6	+/- 0,3	(7)
783	*H. divaricatus*	III	21		(1)	0		(1)	26	+/- 4,6	(3)	0		(2)
290	*H. grosseserratus*	III	10,5	+/- 6	(2)	0		(2)	30,2	+/- 4,9	(5)	0		(5)
1013	*H. tuberosus*	III	22,2	+/- 2,8	(7)	0		(6)	32,1	+/- 4,3	(10)	0,2	+/- 0,1	(9)
774	*H. annuus*	IV	35	+/- 6,1	(6)	49,8	+/- 10,3	(5)	64,3	+/- 11,8	(9)	69,1	+/- 7,6	(8)
826	*H. annuus*	IV	48,3	+/- 8,4	(7)	59,3	+/- 10,2	(7)	69,4	+/- 10,4	(12)	76,4	+/- 11,4	(10)
2008	*H. annuus*	IV	16,2	+/- 1,9	(10)	6,8	+/- 3,1	(9)	23	+/- 2,1	(5)	5,4	+/- 1,3	(5)
654	*H. annuus*	S**	56,4	+/- 14,1	(7)	51,6	+/- 19	(7)	33,8	+/- 22,1	(4)	30,3	+/- 18,2	(4)
2603 control	*H. annuus*	S	109,4	+/- 12,8	(15)	45,9	+/- 5,1	(14)						
XRQ control	*H. annuus*	S	43	+/- 3,9	(37)	52,5	+/- 3,7	(34)						
														

Mean and Standard Error of the Mean (SEM) of the number of attachments and tubercles at 14 and 21 dai with the race E-BOU, in non-treated and treated (GR24 + DCL) rhizotrons. Raw data are available in **Supplementary File 4**.

*: number of rhizotrons in brackets. **: susceptible accessions: #654 as a susceptible wild H. annuus accession, XRQ and 2603 as cultivated H. annuus susceptible controls.

**Table 4:** Variability of the percentage of necrotic tubercles of 18 wild *Helianthus* accessions phenotyped in rhizotrons at 28 dai, using the broomrape race E-BOU.

Accession	Species	Class	Non-treated						Treated(GR24 + DCL)					
			Tubercles-28 dai			% Necrotic tubercles- 28 dai			Tubercles-28dai			% Necrotic tubercles-28 dai		
			mean	SEM	*	mean	SEM	*	mean	SEM	*	mean	SEM	*
588	*H. bolanderi*	I	0	0	(8)	–	–	–	0	0	(1)	–	–	–
2601	*H. exilis*	I	0	0	(8)	–	–	–	0	0	(11)	–	–	–
325	*H. tuberosus*	I	0	0	(8)	–	–	–	0	0	(6)	–	–	–
584	*H. bolanderi*	II	0	0	(5)	–	–	–	1,2	+/-0,4	(6)	20	+/- 14,3	(5)
786	*H. debilis tardiflorus*	II	0,8	+/- 0,4	(5)	0	0	(2)	40	+/-13	(8)	21,2	+/- 8,5	(7)
837	*H. debilis tardiflorus*	II	1,4	+/-0,6	(5)	0	0	(3)	35,1	+/-3,6	(8)	5,1	+/- 2,3	(3)
736	*H. petiolaris*	II	0	0	(10)	–	–	–	3,7	+/-1,6	(7)	4,8	+/- 3,7	(3)
761	*H. petiolaris*	II	1	+/-0,3	(13)	7,1	+/- 5,1	(2)	9	+/-2	(11)	1,1	+/- 0,6	(10)
677	*H. praecox hirtus*	II	0,8	+/- 0,5	(8)	100	0	(1)	100	+/-3,8	(8)	27,5	+/- 10,7	(6)
679	*H.praecox runyonii*	II	0	0	(5)	–	–	–	29	+/-7,1	(10)	32,8	+/- 6,4	(10)
3000	*H. praecox praecox*	II	0,2	+/- 0,1	(7)	100	0	(1)	8	+/-1,8	(7)	20,5	+/- 8,9	(7)
783	*H. divaricatus*	III	0	0	(1)	–	–	–	2	+/-1,4	(2)	100	0	(1)
290	*H. grosseserratus*	III	0	0	(2)	–	–	–	0	0	(5)	–	–	–
1013	*H. tuberosus*	III	0	0	(6)	–	–	–	0	0	(7)	–	–	–
833	*H. annuus*	III	0,4	+/-0,3	(5)	0	0	(1)	0,6	+/- 0,3	(7)	50	+/- 35,4	(2)
774	*H. annuus*	IV	66,8	+/-13	(5)	2,2	+/- 1,1	(5)	75	+/-8,4	(8)	3,8	+/- 1,7	(8)
826	*H. annuus*	IV	64	+/-10,9	(6)	3,1	+/- 1,1	(6)	78,8	+/-11,7	(10)	5	+/- 1,8	(10)
2008	*H. annuus*	IV	6,3	+/-2,7	(9)	1,3	+/- 0,8	(7)	6	+/-1,9	(5)	4	+/- 2,7	(4)
654	*H. annuus*	S**	57	+/-20,8	(7)	0,1	+/- 0,1	(6)	34	+/-19,6	(4)	0	0	(4)
2603 control	*H. annuus*	S	67	+/-6,2	(14)	0,6	+/- 0,3	(14)						
XRQ control	*H. annuus*	S	70	+/-4,8	(32)	1,8	+/- 0,5	(32)						

Mean and Standard Error of the Mean (SEM) of the number of tubercles and percentage of necrotic tubercles at 28dai, with the race E-BOU, in non-treated and treated (GR24 + DCL) rhizotrons. Raw data are available in **Supplementary File 4**.

*: in brackets: number of rhizotrons. **: Susceptible accession and controls.

We classified the accessions in four classes (**Table 5**) depending on the ability to induce broomrape seed germination and to develop tubercle (following GS treatment): (*i*) the accessions belonging to the class I did not induce seed germination nor developed any tubercles after treatment (only incompatible attachments), (*ii*) the accessions of the class II did not induce seed germination but developed tubercles following treatment (mixture of compatible and incompatible attachments), (*iii*) the accessions of class III induced seed germination and incompatible attachments (no tubercles without or with treatment), and (*iv*) the accessions belonging to class IV induced seed germination and developed compatible attachments leading to tubercles (with or without treatment). This class IV gathered the segregating resistant wild *H. annuus* accessions #2008, for which the number of tubercles was very low (7 tubercles at 21 dai in average, compared to ~50 for the susceptible controls or for the susceptible wild *H. annuus* #654), and the segregating wild *H. annuus* accessions with late resistance mechanisms at the emergence stage (#774 and #826). For these 2 accessions, the resistance could not be observed in rhizotrons as the culture duration did not exceed 28 dai (no emergence developed yet).

**Table 5:** Four phenotyping classes in rhizotrons of 18 wild *Helianthus* accessions, using the broomrape race E-BOU.

	Non-Treated rhizotrons (NT)		Treated rhizotrons (T)							
			(GR24 + DCL)							
Class	Attachments	Tubercles	Attachments	Tubercles	Accessions					Number of accessions
I	–	–	+	–	#588 *bolanderi*, #2601 *exilis*, #325 *tuberosus*					3
II	–	–	+	+	#584 *bolanderi*, #*786 debilis tardiflorus*, #*837 debilis tardiflorus*, #736 *petiolaris*, #*761 petiolaris*, #677 *praecox*, #679 *praecox*, #3000 *praecox*,					8
III	+	–	+	–	#783 *divaricatus*, #290 *grosseserratus*, #1013 *tuberosus*, #833 *annuus*					4
IV	+	+	+	+	#774 *annuus*, #826 *annuus*, #2008 *annuus*					3

Eighteen accessions phenotyped in rhizotrons were classified in 4 classes depending on the ability of root exudates to induce Orobanche seed germination, and to develop tubercles (without or with GS treatment (GR24 + DCL).

Statistical analysis showed that treatment with GS did not affect significantly the number of attachments of the accessions from the classes III and IV, nor the number of tubercles of the accessions from the class IV, compared to non-treated rhizotrons. Hence, to compare the number of attachments from the accessions of the 4 classes, only treated rhizotrons were considered. Statistical analysis of the number of attachments at 14 dai showed there was no significative difference between wild accessions for this parameter except for the accession #826 *H. annuus* (**Supplementary File 8A**). These results suggest that once the seeds have germinated, there is no resistant mechanisms at the haustoriogenesis stage in these wild *Helianthus* accessions. By contrast, statistical analysis of the efficiency of the attachments to develop into tubercles, measured by the percentage of tubercles at 21 dai/attachments at 14 dai, reflected as expected the separation between the classes I and III (no tubercles) from the classes II and IV (**Supplementary File 8B**). Accessions from a given wild *Helianthus* species were not specific of a unique class but could belong to various classes, as illustrated for *H. tuberosus* #325 in class I and #1013 in class III, or *H. annuus* #833 in class III and #774 in class IV.

All the accessions for which root exudates did not induced broomrape seed germination in the root exudate assays belonged to classes I and II as expected (**Figure 3** **;** **Table 5**). The accessions #677 *H. praecox* and #786 *H. debilis* for which root exudates showed a very low level of induction belonged to class II. A few attachments and rare tubercles could be observed in some cases for accessions from classes I and II in non-treated rhizotrons, suggesting a low level of seed germination induction (for example for #679 *H. praecox*, **Table 3**). This may be due to an increased concentration of root exudates and consequently of GS in the root vicinity in rhizotrons, allowing a few broomrape seeds to germinate and develop. Interestingly, the 3 accessions (#783 *H. divaricatus*, #290 *H. grosseserratus* and #1013 *H. tuberosus*) showing an intermediate level of seed germination induction in the root exudate assay (**Figure 3**) all gathered in class III. Class III also included the accession #833 *H. annuus* with strong activity exudates.

At 28 dai, the percentage of necrotic tubercles was counted (**Table 4**). In the case of cultivated controls (2603 and XRQ, non-treated rhizotrons) or the susceptible wild *H. annuus* control #654 (non-treated or treated rhizotrons) the percentage of necrotic tubercles at 28 dai was below 2%, slightly comparable to the percentage of necrotic tubercles of class IV accessions (4-5%). The percentage of necrotic tubercles could be much higher for the accessions from class II, reaching 32.8% for #679 *H. praecox.* Interestingly, the percentage of necrotic tubercles was particularly high for the accessions of class III, regarding the rare tubercles developed. Finally, the percentage of necrotic tubercles at 28 dai of all the accessions of the classes II and IV (with the exception of #761 *H. petiolaris*) was differing significantly at the statistical level from the susceptible accessions XRQ, 2603, and the wild *H. annuus* #654 (**Supplementary File 9**). These results suggested that the tubercle necrosis could play a role in the resistance found in wild *Helianthus* of classes II and IV.

### Incompatible attachments showed similar cellular mechanisms among wild *Helianthus*

**Figure 5:**
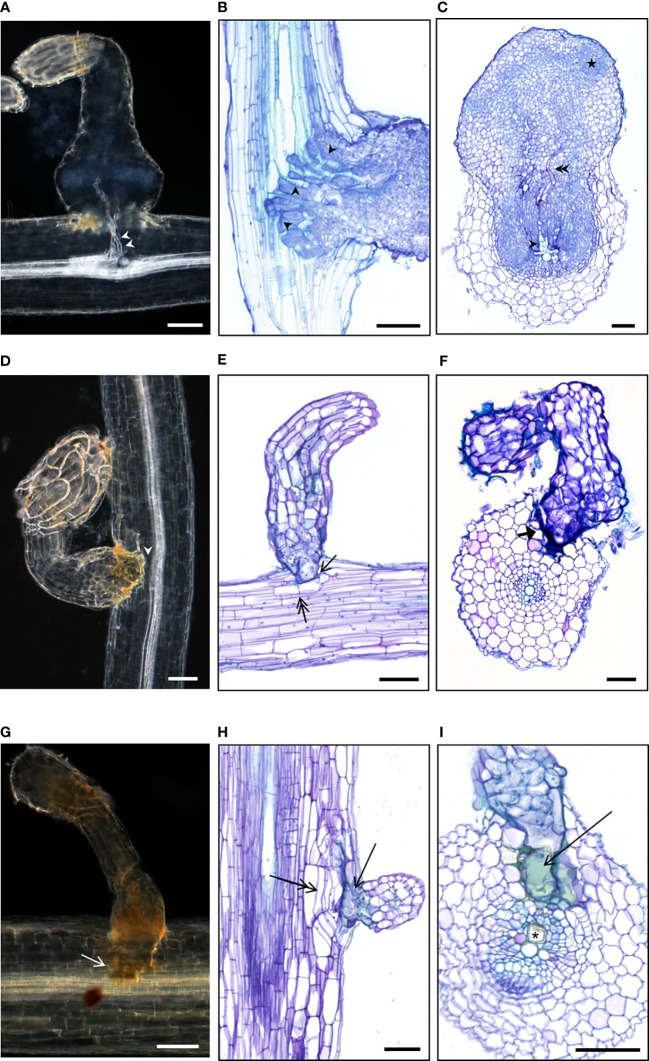
Cytological study of compatible and incompatible attachments revealed various cellular resistant mechanisms **(A–C)** Compatible attachments of susceptible wild *H*. *annuus* or cultivated *H*. *annuus* with established vascular connections. Xylem vessels indicated by white arrowheads in **(A)** and black arrowheads in **(B)** Phloem vessels indicated by double black arrowheads in **(C)** The star indicates *Orobanche* dividing cells preparing bud development. **(D–I)** Incompatible attachments of resistant wild *Helianthus* species. ***(*E, F*)*** no or few cortical cell divisions (double arrow in **E**); phenolic compounds at the basis of the haustorium (arrow in **E**); thick cell wall surrounding the haustorium (thick arrow in **F**). **(H, I)** cortical cell division under or around the haustorium (double arrow in **H**), phenolic compounds (arrow in **H, I**), mucilage deposition in the host xylem vessel (asterisk in **I**). The pictures were taken from various accessions: **(A)** *H. annuus* **#**649; **(B)** sunflower control XRQ; **(C)** sunflower control 2603; **(D)** *H. annuus* **#**833; **(E)** *H. exilis* **#**2601; **(F)** *H. bolanderi***#**584; **(G)** *H. annuus* **#**833; **(H)** *H. grosseserratus* **#**290; **(I)** *H. annuus* **#**833. **(A, D, G)** Whole cleared root samples with attachments. **(B, C, E, F, H, I)** Sections of included root samples with attachments, stained with toluidine blue O. Bars = 100 µm.

To better understand the resistant mechanisms involved at the cell level, during the early stages of the interaction, we performed cytological studies on attachments sampled at 14 dai. In the case of the susceptible control cultivated *H. annuus* or a susceptible wild *H. annuus*, all the attachments at 14 dai were compatible (**Figures 5A–C**): intrusive cells reached the host plant vessels, broomrape vessels were differentiated (phloem and xylem) and vascular connections were established. Consequently, the broomrape attachments swelled thanks to the hijacked nutrient fluxes and small dividing cells were observed on the distal part of the attachments preparing future bud development of the parasitic plant. Accessions from each phenotyping class in rhizotrons were studied (**Supplementary File 10**).

Attachments from the accessions belonging to the classes I and III were all incompatible. Interestingly, cytological studies revealed common cellular patterns whatever the accession used (**Supplementary File 11**). In most cases, incompatible attachments were blocked at the level of the host root cortex (**Figures 5D–F, H**), but sometimes the intrusive cells reached the endodermis (**Figure 5I**). Internal divisions of the host root cells at the tip of the haustorium could be absent (**Figure 5F**), rare (**Figure 5E**) or involving several cell layers (**Figure 5H**). Various cellular patterns were observed and shared among the resistant accessions. Thickness and staining of the cell wall surrounding the haustorium was observed (**Figure 5F**). In most cases, green staining was present revealing phenolic accumulation (Feder and O’Brien, 1968) in the host root cells at the proximity of the haustorium tip, also visible as brownish staining in whole cleared root samples (**Figures 5E, G, H, I**). In addition, modified xylem cells were observed, containing a deposit of gum-like substance as described in the line LR1 by Labrousse et al. (2001). The vascular connections were never established in the case of these incompatible attachments. Swelling of the broomrape was not observed, confirming the absence of nutrient uptake.

For the accessions from classes II, cytological samples were a mix of incompatible and compatible attachments. Incompatible attachments from class II presented the same cell features as the ones described above. In contrast, compatible attachments from class II were similar to compatible attachments observed in the case of the susceptible accessions (**Figure 5A–C**) with established xylem connections. However, in rare cases, green staining in a few cells in proximity of the compatible attachments or modified xylem vessels could be observed, revealing cell defense reactions that could be responsible for subsequent tubercle necrosis as for *H. praecox* (**Supplementary File 12**). Finally, in the case of the accession #826 *H. annuus* from class IV, compatible attachments were similar to the ones of susceptible accessions, and no defense reaction was observed at the cellular level, at 14 dai.

## Discussion

In this study, we have characterized the GS perception of 5 broomrape races. The GS perception variability among these races confirmed the presence of various GS perception modalities in the *O. cumana* species, potentially related to KAI2 receptor diversity (Nelson, 2021). Sunflower root exudates have been shown to contain the sesquiterpene lactones DCL and costunolide (Raupp and Spring, 2013; Rial et al., 2021), as well as the strigolactone heliolactone (Ueno et al., 2014). It would be interesting to confirm the contrasted perception of these 5 races, using heliolactone, which synthesis is in progress.

We have screened a large collection of wild *Helianthus* accessions at various stages of the interaction with broomrape. The screening in pots showed the strong potential of wild *Helianthus* for broomrape resistance, even to the highly virulent race G populations, as reviewed by Seiler and Jan (2014), and particularly in *Helianthus* species other than *H. annuus* (14 out of 15 wild species were resistant). Only the 2 accessions of *H. argophyllus* were completely susceptible, in contrast to previous results using other accessions from this species (Christov et al., 2009; Petcu and Pacureanu, 2012; Terzic et al., 2020). Root exudate assays showed that there was a strong induction of germination for the 4 accessions of *H. annuus* segregating for the resistance, similar to the susceptible accessions from *H. argophyllus* or the sunflower control XRQ. By contrast, a large majority of wild *Helianthus* species other than *H. annuus* did not induce *O. cumana* seed germination (11 accessions out of 14). Since treatment of rhizotrons with GS was sufficient to induce seed germination and subsequent attachment to the roots of these accessions, this suggests that the absence of germination observed in germination assays with exudates was related to low GS concentrations rather than to the presence of germination inhibitors. Identification and quantification of GS in these exudates would test this hypothesis. Rial et al. (2021) showed that there were various amounts of GS in resistant sunflowers but did not correlate directly GS amounts with seed germination assays. Resistances to parasitic plants, due to the absence of seed germination induction in exudates have been described for various species and their respective parasitic plant, such as sorghum and *Striga* (Gobena et al., 2017), rapeseed and *Phelipanche ramosa* (Gauthier et al., 2012) or tomato and *P. aegyptiaca* (Dor et al., 2011). However, there is only very few report for sunflower and broomrape. One can cite the work of Labrousse et al. (2001) who showed that the low germination level induced by exudates from the resistant line LR1 (derived from *H. debilis)* could partly explain the low number of attachments observed. Thus, the introgression into sunflower lines of resistance genes derived from wild *Helianthus* species, affecting broomrape seed germination, would be extremely valuable.

Screening at the attachment and tubercle stages in rhizotron experiments revealed among the accessions different phenotypic classes according to their ability to induce seed germination and develop tubercles. Interestingly, when the rhizotrons were treated with GS, attachments always developed and the number of attachments among the accessions did not differ significantly at the statistical level except for 2 accessions (the sunflower control 2603 and the wild *H. annuus* #826). These results suggest that there are no resistance mechanisms impeding haustorium development among the 18 accessions tested in this study. While costunolide has been shown to be involved in chemotropism of the radicle toward the host root (Krupp et al., 2021), haustorium-inducing factors (HIFs) have not been identified in the sunflower-broomrape interaction to date. This is in contrast to rapeseed and *P. ramosa* (Goyet et al., 2019), for which cytokinin has been shown to induce haustoriogenesis. Incompatible attachments (IA) were observed in accessions belonging to classes I, II and III. Interestingly, it appeared that multiple mechanisms of resistance were present in one accession (as for example for the accession #325 *H. tuberosus*, with the absence of germination induction and absence of tubercle development following GS treatment). It is interesting to note that while the two *H. debilis* subsp. *tardiflorus* tested in this study did not induce germination, the exudates of the introgressed line DEB2 (with the resistance gene *Or_DEB2_* introgressed from *H. debilis* subsp. *tardiflorus*) induced germination (Fernandez-Aparicio et al., 2022). This was probably because this specific resistance mechanism was not evaluated in the selection and introgression process aimed at developing the DEB2 line, in which only emerged broomrapes were phenotyped.

Cytological studies of incompatible attachments revealed that similar cellular mechanisms were observed whatever the *Helianthus* accession. In most cases, the parasitic plant was blocked in the host cortex, as reported by Dörr et al. (1994) and rarely reached the endodermis. The cell mechanisms leading to incompatible attachment, were the presence of an encapsulation layer surrounding the haustorium, a phenolic accumulation at the basis of the haustorium suggesting host defense reactions, and host xylem modification with a gum-like substance deposition. Interestingly, all these cellular mechanisms have already been reported for sunflower broomrape resistance (Dörr et al., 1994; Labrousse et al., 2001; Echevarria-Zomeño et al., 2006; Duriez et al., 2019). These results suggest that defense reactions against broomrape rely on a set of cellular mechanism common to *Helianthus* species. Whether these cellular defense responses are shared by other sunflower/root pathogen interactions as well, remain to be defined. For the accessions belonging to the phenotyping class II, there was a mixture of incompatible (no tubercle development) and compatible attachments (leading to tubercle development). Some of these tubercles became necrotic, revealing later resistant mechanisms. The percentage of necrotic tubercles increased between 21 dai and 28 dai, suggesting that the duration of 28 dai in rhizotrons may not be sufficient to achieve the observation of this resistant phenotype. It would be interesting to increase the duration of the culture for these accessions. Necrotic tubercles have been described by Labrousse et al. (2001) in the resistant line LR1, derived from *H. debilis.* In this study, this phenotype was present in the species *H. petiolaris, H. praecox* and *H. bolanderi* in addition to *H. debilis*. Tubercle necrosis could result from similar but delayed cellular mechanisms as the ones observed for incompatible attachments: phenolic accumulation and host xylem modifications, but also from other mechanisms yet to be identified. Finally, resistance at the late emergence stage resulting in the arrest of the emergence growth was observed for the wild *H. annuus* accessions #774 and #826. This resistance phenotype looked similar to the one controlled by *Or_SII_* (Martin-Sanz et al., 2020), for which the wild origin was not published.

In most cases, regardless of the screening method, no major difference in sunflower resistance was observed between the 2 broomrape populations used. We then investigated whether the resistances observed in wild *Helianthus* species were effective using 3 other highly virulent broomrape populations. For this purpose, we selected 7 accessions from classes I to III showing resistance at early stages and performed a phenotyping assay in pots using 3 races G from various countries (Turkey, Spain, and Russia) in addition to the Romanian G population used in the first screenings (**Supplementary File 13**). This additional assay (No. 3) showed that at least 3 accessions (#2601 *H. exilis*, #325 *H. tuberosus* and #584 *H. bolanderi*) were completely resistant to the 4 highly virulent races G and confirmed the very high potential of wild *Helianthus* resistances to control the highest virulent races.

The wild *Helianthus* species have been collected in North America (Terzic et al., 2020) where sunflower broomrape is not present. It raises thenceforth, the question of whether the resistant genes present in the wild *Helianthus* originally evolved for resistance to other pathogens. Interestingly, 3 resistant genes to parasitic plants identified so far coded for receptor-like proteins: in cowpea (resistance to *Striga*; Li and Timko, 2009), in tomato (resistance to *Cuscuta reflexa*; Hegenauer et al., 2016) or in sunflower for broomrape resistance (Duriez et al., 2019). It is thus tempting to make the hypothesis that some of the broomrape resistance genes present in wild *Helianthus* species could be also receptor-like proteins. However, it is not clear how resistant genes to various pathogens might be closely related as has been suggested for rust and mildew (Bachlava et al., 2011). In this study, the fact that the 7 wild *H. annuus* accessions originally selected for resistance to downy mildew (#358, #650, #662, #775, #963, #999, and Idaho; Pecrix et al., 2018) were susceptible to broomrape does not support this hypothesis. Furthermore, could random mutations/duplications in genes coding for receptors in wild *Helianthus* lead to resistance to broomrape?

In conclusion, we showed that resistances in wild *Helianthus* species affected various stages of the interaction: absence of broomrape seed germination induction, incompatible attachment, tubercle necrosis and arrested growth of emerged stems. Thus, these species represent a valuable resource of resistance genes to build a sustainable pyramidal resistance to broomrape in sunflower like the successive defenses of a fortress. These wild *Helianthus* species can be used for pre-breeding resources, through interspecific hybridization (Warburton et al., 2017). Interspecific hybridization have been reported successfully for most of the wild species used in this study: for the annual wild *Helianthus* species *argophyllus, bolanderi, debilis*, *petiolaris, praecox* (reviewed by Warburton et al., 2017), and even for the perennial wild species *grosseseratus, pauciflorus, tuberosus*, *divaricatus, nuttallii* and *strumosus* (reviewed by Christov et al., 2009; Terzic et al., 2010; Warburton et al., 2017; and Sukno et al., 1999). This shows that the introgression of resistance genes from wild *Helianthus* species into sunflower varieties is possible. Moreover, introgressed line (IL) populations have already been produced in previous projects for the accessions #833, #2008, #774 and #826 from *H. annuus*, and IL phenotyping in broomrape infested fields should lead to the identification of new resistant genes in the near future. Finally, lines derived from interspecific hybridization could be of interest for other breeding programs (reviewed in Skoric, 2016), as shown for salinity tolerance (*H. paradoxus*) and drought tolerance (*H. argophyllus*), or for resistances to other pathogens such as downy mildew, rust, *Phoma*, and *Macrophomina phaseolina* in the case of *H. tuberosus*.

## Data availability statement

The original contributions presented in the study are included in the article/**Supplementary Material**. Further inquiries can be directed to the corresponding author.

## Author contributions

MC designed the study, coordinated the project, performed experiments, analyzed the results and wrote the manuscript. M-CA performed cytological experiments. M-CB produced the resources (*Helianthus* and broomrape). SD performed exudate experiments. TF and AL performed rhizotrons experiments. FJ performed statistical analyses. BP-V, J-BP, and LV performed experiments, analyzed the data and participated to the construction of the manuscript. PD and SM designed the study, assisted with the construction and the writing of the manuscript. All authors contributed to the article and approved the submitted version.

## Funding

This study was performed in the frame of a 3-year project (ResODiv), funded by “Promosol” (the association of French Sunflower and Rapeseed Breeders for promoting these crops).

## Acknowledgments

We thank L. Riesberg for sending seeds of some of the wild *Helianthus* accessions used in this study. This study was supported by the “Laboratoires d’Excellences (LABEX)” “Towards a Unified theory of biotic interactions: roLe of environmental Pertubations” (TULIP; ANR-10-LABX-41)” and/or by the “École Universitaire de Recherche (EUR)” TULIP-GS (ANR-18-EURE-0019).

## Conflict of interest

The authors declare that the research was conducted in the absence of any commercial or financial relationships that could be construed as a potential conflict of interest.

## Publisher’s note

All claims expressed in this article are solely those of the authors and do not necessarily represent those of their affiliated organizations, or those of the publisher, the editors and the reviewers. Any product that may be evaluated in this article, or claim that may be made by its manufacturer, is not guaranteed or endorsed by the publisher.
