# Supplementary material for: Wild Helianthus species: A reservoir of resistance genes for sustainable pyramidal resistance to broomrape in sunflower
Source: Front Plant Sci. 2022 Oct 20;13:1038684. doi: 10.3389/fpls.2022.1038684 (PMC9630478; doi:10.3389/fpls.2022.1038684)

Suppl. File 1. List of wild *Helianthus* accessions used in this study and associated phenotyping assays.

Seventy one accessions were phenotyped in 6 L pots: 36 wild *H. annuus* accessions and 35 wild *Helianthus* accessions from other species than *annuus*, including 21 annual accessions from 8 species (12 taxa) and 14 perennial accessions from 7 species. Eighteen of these accessions were phenotyped for the exudate activity on broomrape seed germination and in rhizotrons.

				**Phenotyping**		
**code**	***Helianthus* species**	**PI**	**Annual/ (A) Perennial (P)**	**6 L pots Emergence stage**	**Exudate activity on seed germination**	**Rhizotrons attachment and tubercle stage**
351	*annuus*		A	x		
358	*annuus*		A	x		
421	*annuus*		A	x		
649	*annuus*		A	x		
650	*annuus*		A	x		
654	*annuus*		A	x		x
661	*annuus*		A	x		
662	*annuus*		A	x		
774	*annuus*		A	x	x	x
775	*annuus*		A	x		
826	*annuus*		A	x	x	x
829	*annuus*		A	x		
831	*annuus*		A	x		
833	*annuus*		A	x	x	x
928	*annuus*		A	x		
955	*annuus*		A	x		
963	*annuus*		A	x		
980	*annuus*		A	x		
999	*annuus*		A	x		
1016	*annuus*		A	x		
Idaho	*annuus*		A	x		
2000	*annuus*	PI 413021	A	x		
2001	*annuus*	PI 413095	A	x		
2002	*annuus*	PI 413131	A	x		
2003	*annuus*	PI 435368	A	x		
2004	*annuus*	PI 435457	A	x		
2005	*annuus*	PI 435531	A	x		
2007	*annuus*	PI 435850	A	x		
2008	*annuus*	PI 468571	A	x	x	x
2010	*annuus*	PI 586809	A	x		
2011	*annuus*	PI 586819	A	x		
2012	*annuus*	PI 586879	A	x		
2013	*annuus*	PI 592312	A	x		
2014	*annuus*	PI 613752	A	x		
2015	*annuus*	PI 613783	A	x		
2016/2017	*annuus*	PI 649814	A	x		

code	*Helianthus* species	PI	Annual/ (A) Perennial (P)	Phenotyping<br>6 L pots Emergence stage	Exudate activity on seed germination	Rhizotrons attachment and tubercle stage
525/ 2100	*anomalus*	PI 468638	A	x		
861	*argophyllus*		A	x	x	
2202	*argophyllus*	PI 435629	A	x		
255	*bolanderi*		A	x		
584	*bolanderi*		A	x	x	x
588	*bolanderi*		A	x	x	x
2301	*bolanderi*	PI 435641	A	x		
2400	*debilis cucumerifolius*	PI 653609	A	x		
835	*debilis debilis*	PI 435671	A	x		
786	*debilis tardiflorus*	PI 468691	A	x	x	x
837	*debilis tardiflorus*	PI 468689	A	x	x	x
2600	*exilis*	PI 649895	A	x		
2601	*exilis*	PI 664629	A	x	x	x
2700	*neglectus*	PI 435768	A	x		
2701	*neglectus*	PI 597916	A	x		
736	*petiolaris petiolaris*	PI 468823	A	x	x	x
761	*petiolaris petiolaris*		A	x	x	x
198	*praecox hirtus*		A	x		
677	*praecox hirtus*	PI 468850	A	x	x	x
3000	*praecox praecox*	PI 468851	A	x	x	x
679	*praecox runyonii*	PI 468860	A	x	x	x
232	*divaricatus*	PI 435675	P	x		
783	*divaricatus*	PI 435675	P	x	x	x
290	*grosseserratus*		P	x	x	x
1014	*grosseserratus*		P	x		
1036	*grosseserratus*		P	x		
239	*nuttallii*	PI 435779	P	x		
1217	*nuttallii*	PI 531047	P	x		
926	*pauciflorus subrhomboideus*		P	x		
969	*pauciflorus subrhomboideus*		P	x		
1031	*pauciflorus subrhomboideus*		P	x		
1225	*strumosus*		P	x		
325	*tuberosus*		P	x	x	x
1013	*tuberosus*		P	x	x	x
3100	*winteri*	PI 673292	P	x		

Suppl. File 2. Timeline of the culture and phenotyping in rhizotrons.

Phenotyping in rhizotrons of the wild *Helianthus* species and the control cultivated *H. annuus* was performed at the attachment stage (14 days after inoculation-dai), tubercle stage (21 dai) and necrotic tubercle stage (28 dai). At 14 dai, samples (fragments of roots with compatible or incompatible attachments) were prepared for cytological studies. D0: Day 0 is the day of inoculation.

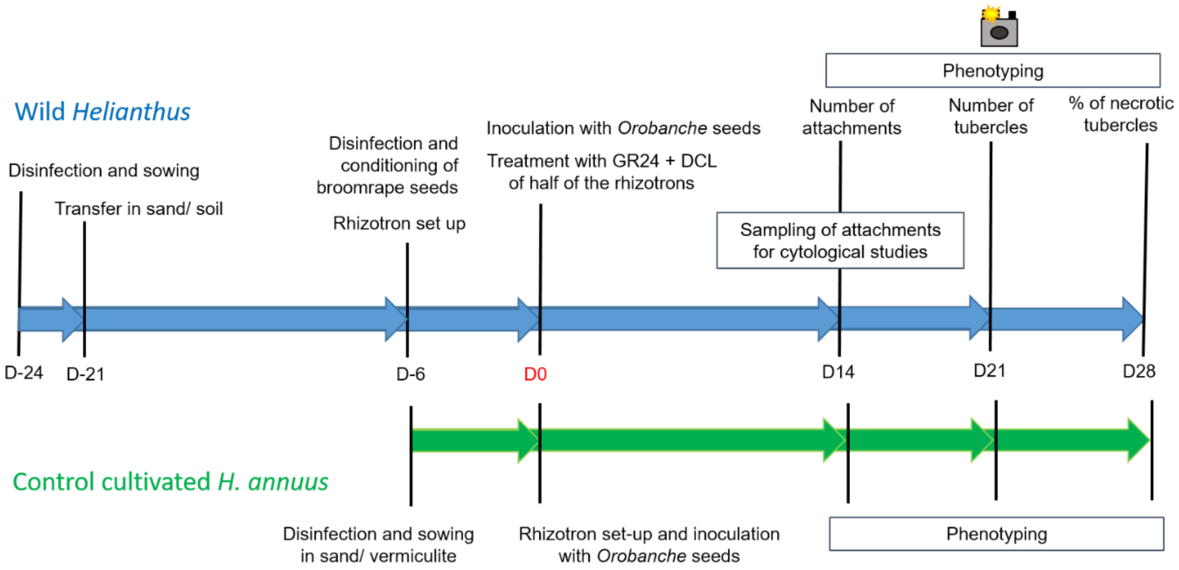

Suppl. File 3. Numbers of rhizotrons and independent experiments/ accessions.

Rhizotrons were inoculated with the race E-BOU (2017). At least 2 independent experiments were performed for each accession. The numbers of Non-Treated (NT) and Treated (T) rhizotrons (with GR24 + DCL) were variable depending on the germinating ability of the accession. At 14 dai and 21 dai, the number of attachments and tubercles were counted respectively. As one rhizotron/ experiment was used for cytological sampling at 14 dai, the number of rhizotrons differed between 14 and 21 dai.

		**Number of rhizotrons**				
**code**	***Helianthus* species**	**14 dai-NT**	**14 dai- T**	**21 dai-NT**	**21 dai-T**	**number of independent experiments**
XRQ	*annus*	39	0	39	0	12
2603	*annuus*	13	0	13	0	5
654	*annuus*	7	4	7	4	2
774	*annuus*	6	9	5	8	2
826	*annuus*	7	12	6	10	3
833	*annuus*	5	8	5	7	2
2008	*annuus*	10	5	10	5	2
584	*bolanderi*	5	7	5	6	2
588	*bolanderi*	8	2	8	2	4
786	*debilis tardiflorus*	5	8	5	8	2
837	*debilis tardiflorus*	12	9	12	8	3
783	*divaricatus*	1	3	1	2	2
2601	*exilis*	8	12	8	12	3
290	*grosseserratus*	2	5	2	5	2
736	*petiolaris petiolaris*	10	7	10	7	4
761	*petiolaris petiolaris*	14	11	14	10	4
677	*praecox hirtus*	8	9	8	9	2
679	*praecox runyonii*	12	10	12	10	4
3000	*praecox praecox*	6	7	6	7	2
325	*tuberosus*	8	7	8	6	3
1013	*tuberosus*	5	10	6	9	2

Suppl. File 4. Raw data of the number of attachments (at 14 dai), tubercles (at 21 dai) and necrotic tubercles (at 28 dai) / rhizotron for each accession.

Raw data are detailed in the specific joined file. NA: data Not Available.

Wild *Helianthus* plantlets were inoculated with conditioned *Orobanche* seeds (race E-BOU), following 15 days of culture in soil and 6 days in rhizotrons (see Materials and Methods and Suppl. File 2) except for the following experiments: I15: 17 days of culture in soil, and inoculation the day of transfer in rhizotron; I16 and I18: inoculation the day of transfer in rhizotron; I19: plantlets were grown 27 days in soil and 6 days in rhizotron before inoculation.

.

Suppl. File 5. Germination dose-response curves of various *O. cumana* populations to various germination stimulants.

Germination dose-response curves were modelled after normalization of the germination activity (bar : ± SD) thanks to the germination percentage of each populations obtained with 1 µM GR24 (**Fig. 1a**) using a four parameter logistic curve. For each compound, (±)-GR24; DCL and costunolide, and equimolar mixtures, a range of concentrations from 1 µM to 0.1 pM were applied to conditioned seeds of five *O. cumana* populations.

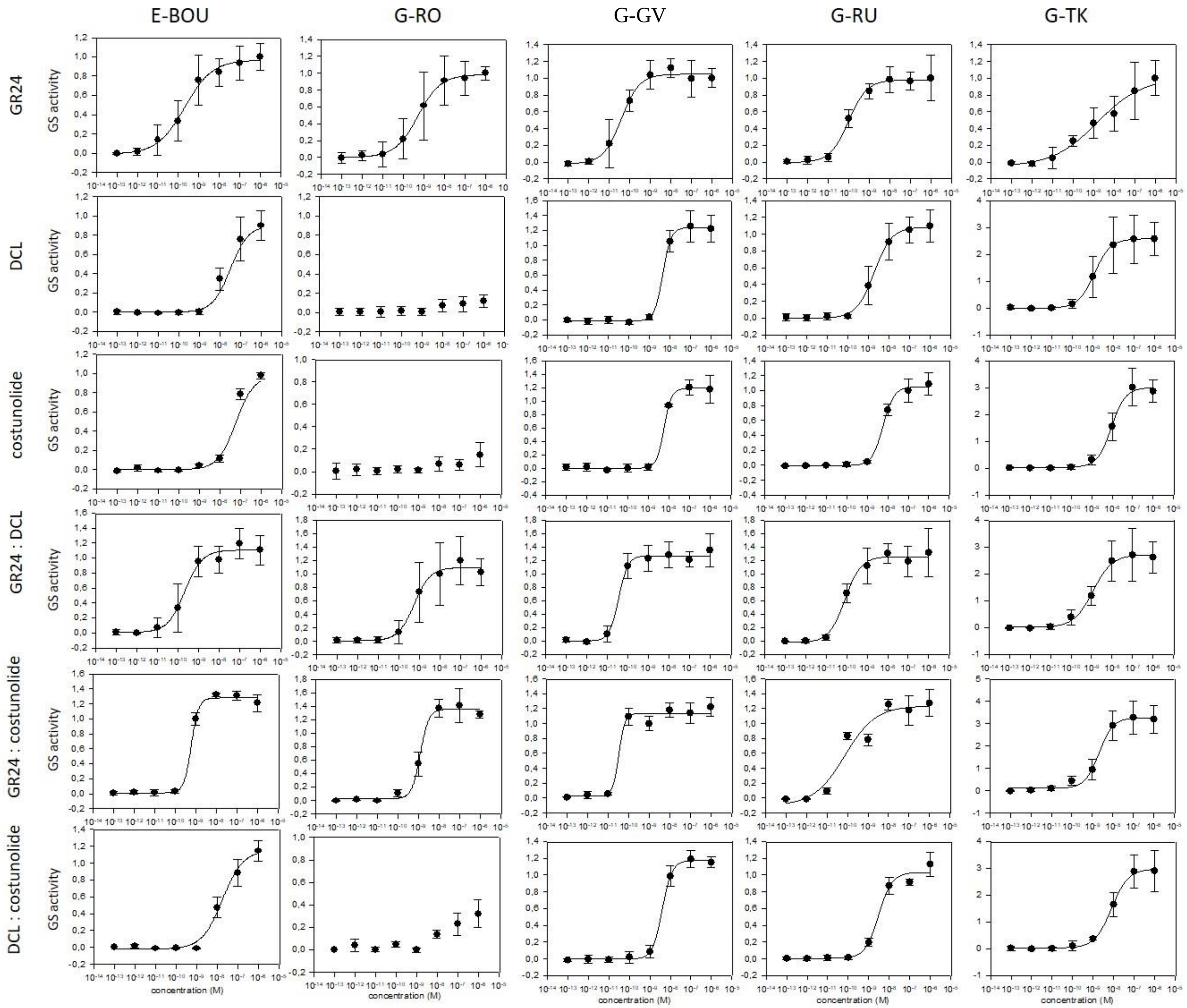

Suppl. File 6. Half-maximal effective concentration (EC50) of various germination stimulants on various *O. cumana* populations.

EC50 was determined for every compounds and mixtures, and for the five O. cumana populations thanks to the generated dose-response curves presented in the Suppl. File 5. Bar : ± SE. Different letters indicate significant differences at $p < 0.05$ (Student-Newman-Keuls Methodtest) between germination stimulants for a population (corresponding colored letters) or between population for a germination stimulant (black letters). Due to poor germination in response to DCL and costunolide, the EC50 of the G-RO population could not be determined.

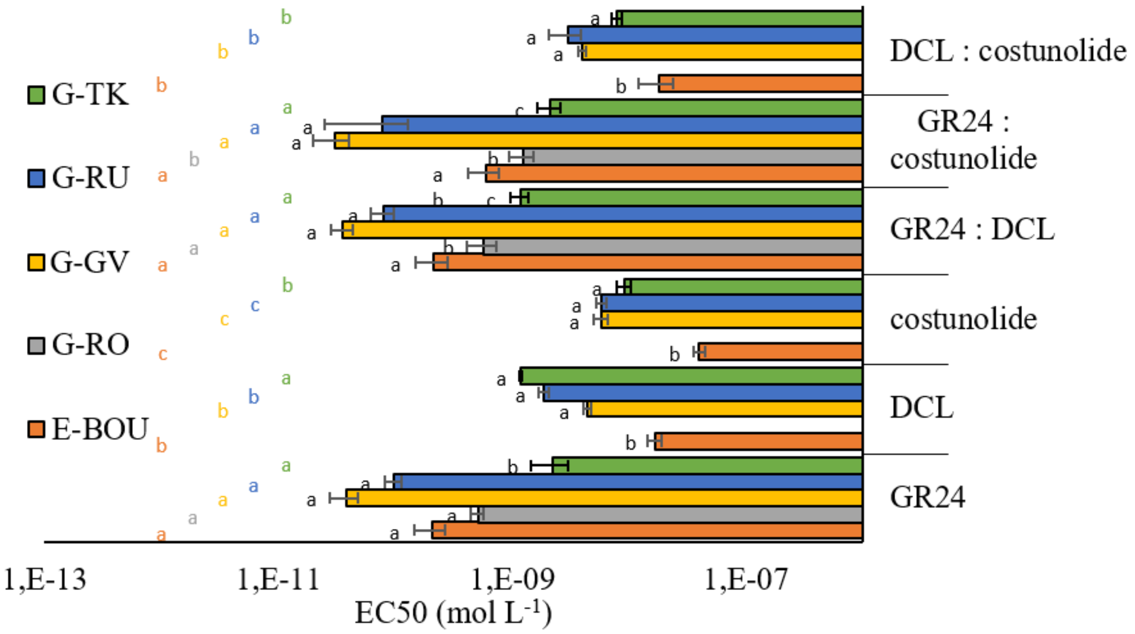

Suppl. File 7. Kinetic of GS activity exuded by a set of *Helianthus* accessions.

Germination activities were normalized thanks to the germination percentage of both population obtained with GR24:DCL (equimolar, 1µM). For each accessions (except #774a and #826a), root exudates collected 3, 4, 5 and 6 weeks after sowing were applied to conditioned seeds of the two populations, E-BOU and G-RO. For #774a and #826a root exudates were collected at 3 and 4 weeks of culture only. Bar : ± SE**.**

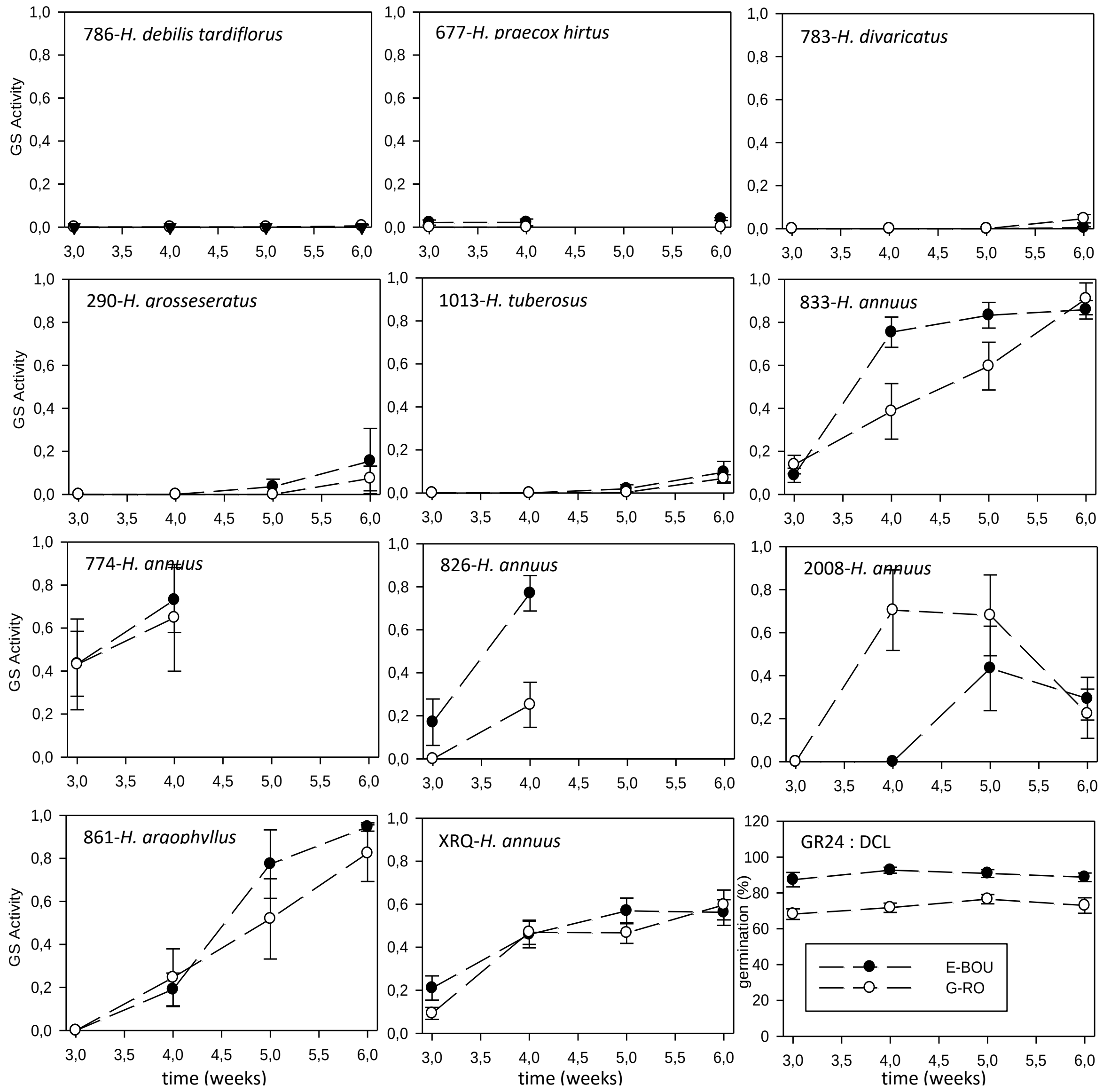

Suppl. File 8. Quantitative analysis of the phenotyping data at early stages (attachments and tubercles) in rhizotrons inoculated with the race E-BOU.

Data were analysed taking into account only GS-treated rhizotrons, except for the cultivated susceptible controls XRQ and 2603. **8a.** There was no significant statistical difference in the number of attachments at 14 dai between the wild resistant accessions except for the wild *H. annuus* #826a. **8b.** Ordering the accessions by the efficiency of the development of attachments into tubercles (% of tubercle/ attachment) revealed a significant difference between phenotyping classes I and III, compared to the classes II and IV.

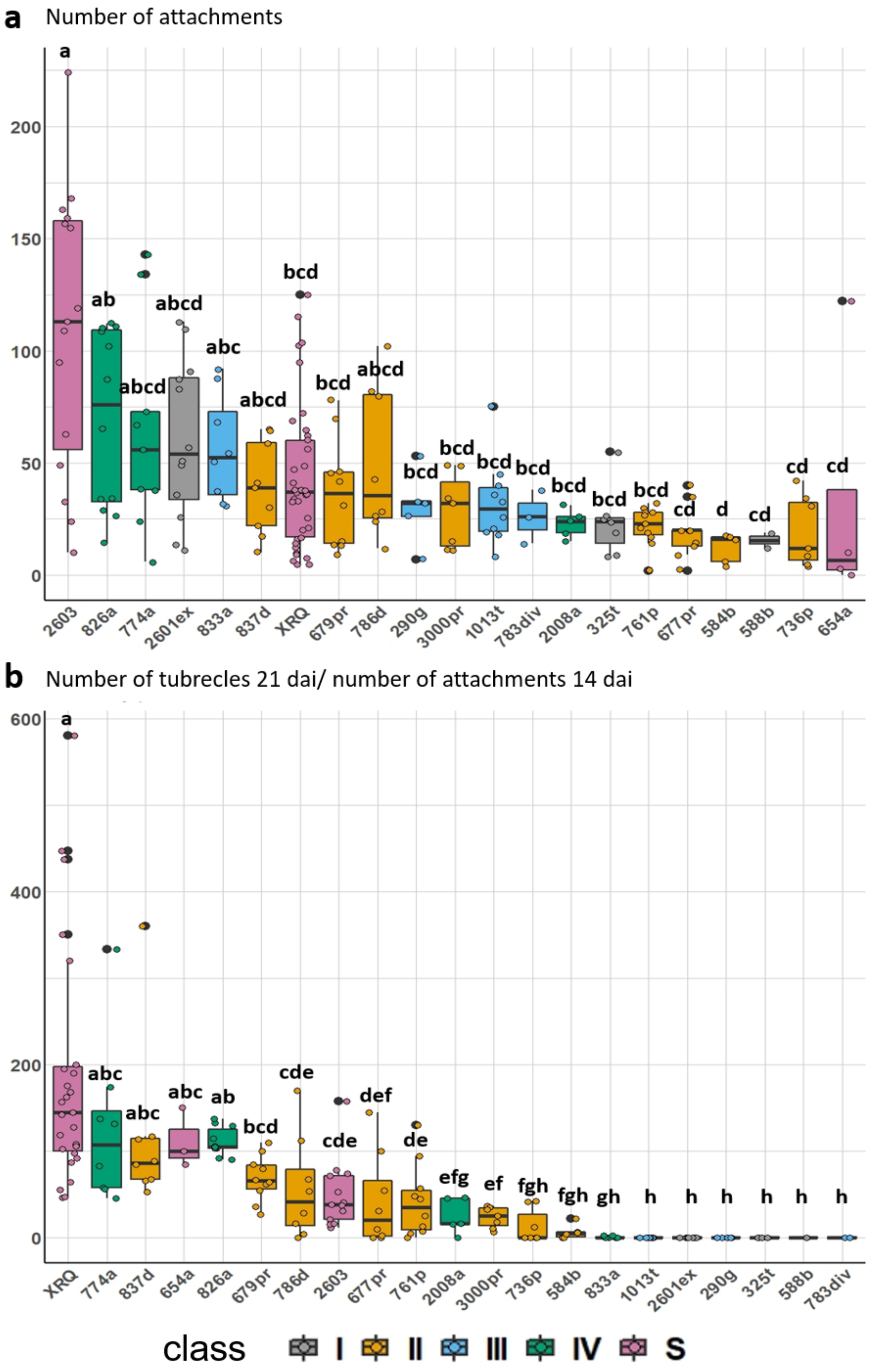

Suppl. File 9. Quantitative analysis of the percentage of necrotic tubercles at 28 dai in rhizotrons inoculated with the race E-BOU.

Data were analysed taking into account only GS-treated rhizotrons, except for the cultivated susceptible controls XRQ and 2603 (non-treated rhizotrons). Accessions without tubercle development were not taken into account (Class I and Class III, except #833a which develop few tubercles). Statistical analysis was performed using the Kruskal-Wallis test (α = 0.05).

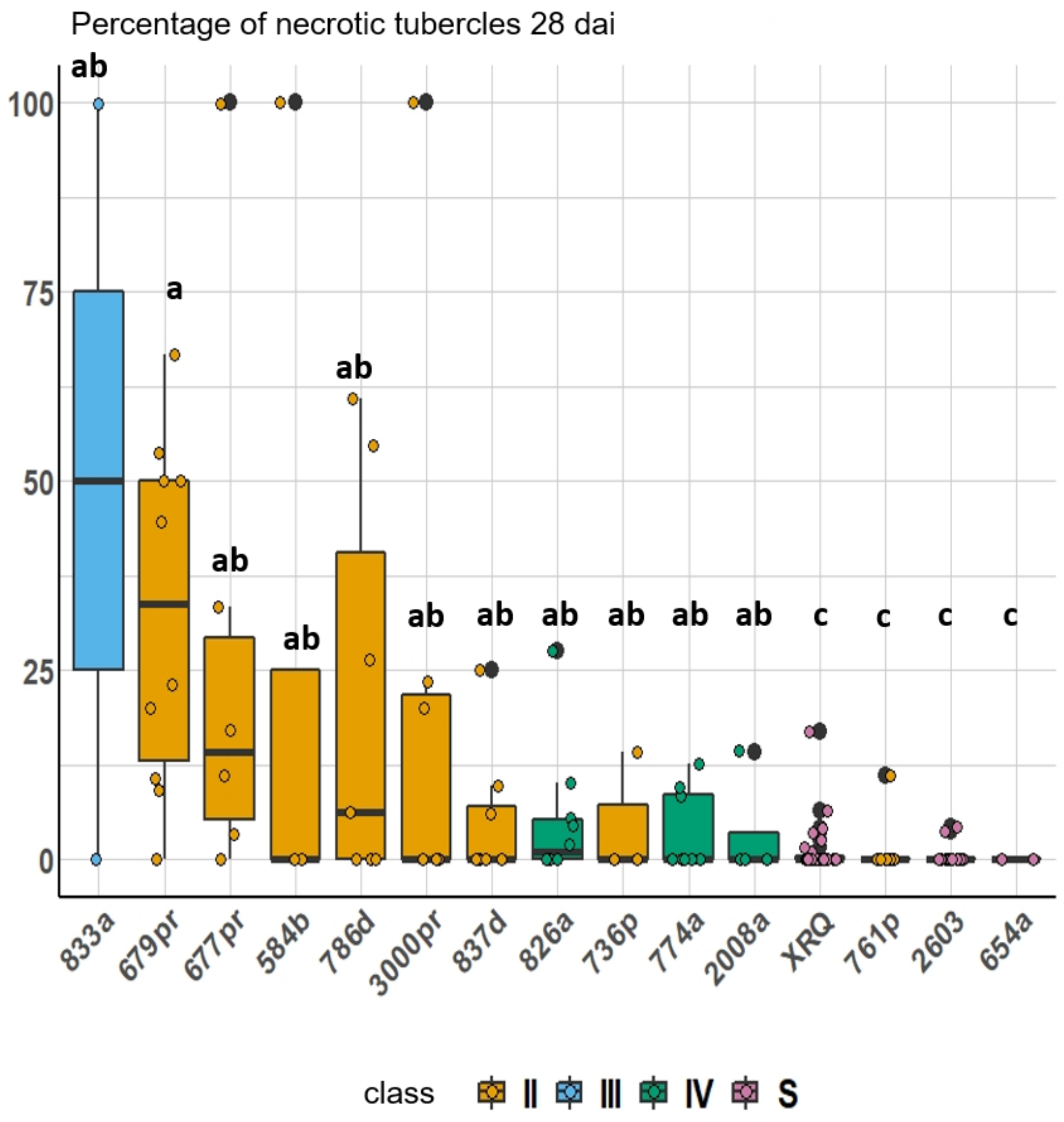

Suppl. File 10. List of wild *Helianthus* accessions used for cytological studies and number of observed samples for each accession.

A few accessions from each phenotyping class in rhizotrons were used, inoculated with the race E-BOU. Whole root segment with attachment were cleared with chloral hydrate, or sectioned, imbedded in technovit system and stained with toluidine blue O. Attachments of accessions from classes I and III were all Incompatible (IA). Attachments of accessions from class IV were all Compatible (CA). For the accessions from Class II there were a mixture of IA and CA. As accessions from Classes I and III did not induce seed germination, attachments were sampled only from GS-treated rhizotrons for these accessions.

		Class	Treatment		cleared samples		sectioned samples	
accession	species	(phenotyping in rhizotrons)	No treatment (NT) or Treatment (T)	attachment type incompatible (IA) compatible (CA)	number of samples	number of attachments	number of samples	number of attachments
2601	*exilis*	I	T	IA	11	40 IA	7	12 IA
325	*tuberosus*	I	T	IA	4	6 IA	5	13 IA
584	*bolanderi*	II	T	IA/ CA	4	5 IA	6	8 IA/ 2CA
786	*debilis tardiflorus*	II	T	IA/ CA	4	5 IA/ 3 CA	4	5 IA/ 3 CA
736	*petiolaris*	II	T	IA/ CA	7	10 IA/ 3 CA	6	3 IA/ 3 CA
761	*petiolaris*	II	T	IA/ CA	3	1 IA/ 2 CA	6	10 IA/ 10 CA
679	*praecox runyonii*	II	T	CA	3	4 CA	5	8 CA
3000	*praecox praecox*	II	T	IA/ CA	2	1 IA/ 1 CA	4	1 IA/ 6 CA
783	*divaricatus*	III	T	IA	5	5 IA	5	6 IA
290	*grosseserratus*	III	T	IA	3	7 IA	4	7 IA
1013	*tuberosus*	III	NT	IA	6	13 IA	5	6 IA
			T	IA	6	14 IA	5	6 IA
833	*annuus*	III	NT	IA	1	3 IA	1	4 IA
			T	IA	7	15 IA	2	2 IA
826	*annuus*	IV	T	CA	10	19 CA	5	7 CA

Suppl. File 11. Summary of the the cellular phenotypes observed by cytology on attachments (14 dai).

Common cellular resistant mechanisms led to incompatible attachments (IA) independently of the phenotyping classes in rhizotrons. Rarely, some defence reactions were observed in compatible attachments (CA).

**Incompatible attachments (IA)**	**Phenotyping class**	**accessions/ *Helianthus* species**
thick stained cell wall around the haustorium	I	2601 *H. exilis;* 325 *H. tuberosus*
	II	584 *H. bolanderi;* 761 *H. petiolaris;* 3000 *H. praecox praecox*
	III	290 *H. grosseserratus;* 1013 *H. tuberosus*
phenolic accumulation in outer cortex	I	2601 *H. exilis;* 325 *H. tuberosus*
	II	584 *H. bolanderi;* 786 *H. debilis tardiflorus;* 736 *H. petiolaris;* 761 *H. petiolaris;* 3000 *H. praecox praecox*
	III	783 *H. divaricatus;* 290 *H. grosseserratus;* 1013 *H. tuberosus;* 833 *H. annuus*
division in internal host root tissue	all classes	observed in all the 13 accessions
deepest host root tissue reached by intrusive cells:		
outer cortex	I	2601 *H. exilis;* 325 *H. tuberosus;* 584 *H. bolanderi*
	II	786 *H. debilis tardiflorus;* 761 *H. petiolaris;* 3000 *H. praecox praecox*
	III	783 *H. divaricatus;* 290 *H. grosseserratus;* 1013 *H. tuberosus*
inner cortex	I	325 *H. tuberosus*
	II	736 *H. petiolaris;* 761 *H.petiolaris*
	III	1013 *H. tuberosus*
endodermis	I	Not observed
	II	761 *H. petiolaris*
	III	290 *H. grosseserratus;* 833 *H. annuus*
xylem vessels	I	Not observed
	II	736 *H. petiolari;* 761 *H. petiolaris*
	III	833 *H. annuus*
mucilage in xylem vessels	I	584 *H. bolanderi*
	II	761 *H. petiolaris;* 3000 *H. praecox praecox*
	III	833 *H. annuus*
vascular connection	not observed	

**Compatible attachments (CA)**	**Phenotyping class**	**accessions/ *Helianthus* species**
division in internal host root tissue, vascular xylem connection and swelling of the haustorium	I	584 *H. bolanderi* (rare CA)
	II	786 *H. debilis tardiflorus;* 736 *H. petiolaris;* 761 *H. petiolaris;* 679 *H. praecox runyonii;* 3000 *H. praecox praecox;*
	IV	826 *H. annuus*
thick stained cell wall around the haustorium	not observed	
phenolic accumulation in outer cortex	II	679 *H. praecox runyonii;* 3000 *H.praecox praecox* (in rare cases)
mucilage in xylem vessels	II	3000 *H. praecox praecox* (in rare cases)

Suppl. File 12. Defence reactions at proximity of compatible attachments revealed by cytological study of Class II accessions.

At 14 dai, accessions from class II, such as *H. praecox*, developed a mixture of incompatible attachments and compatible attachments. In some cases, defence reactions at proximity of these compatible attachments were observed as green staining suggesting phenolic compounds **(a.** accession #677 *H. preacox*; arrowheads)**,** or gum-like substance in xylem vessels **(b.** accesion #679 *H. praecox*; red asterisks). Bar = 100 µ.

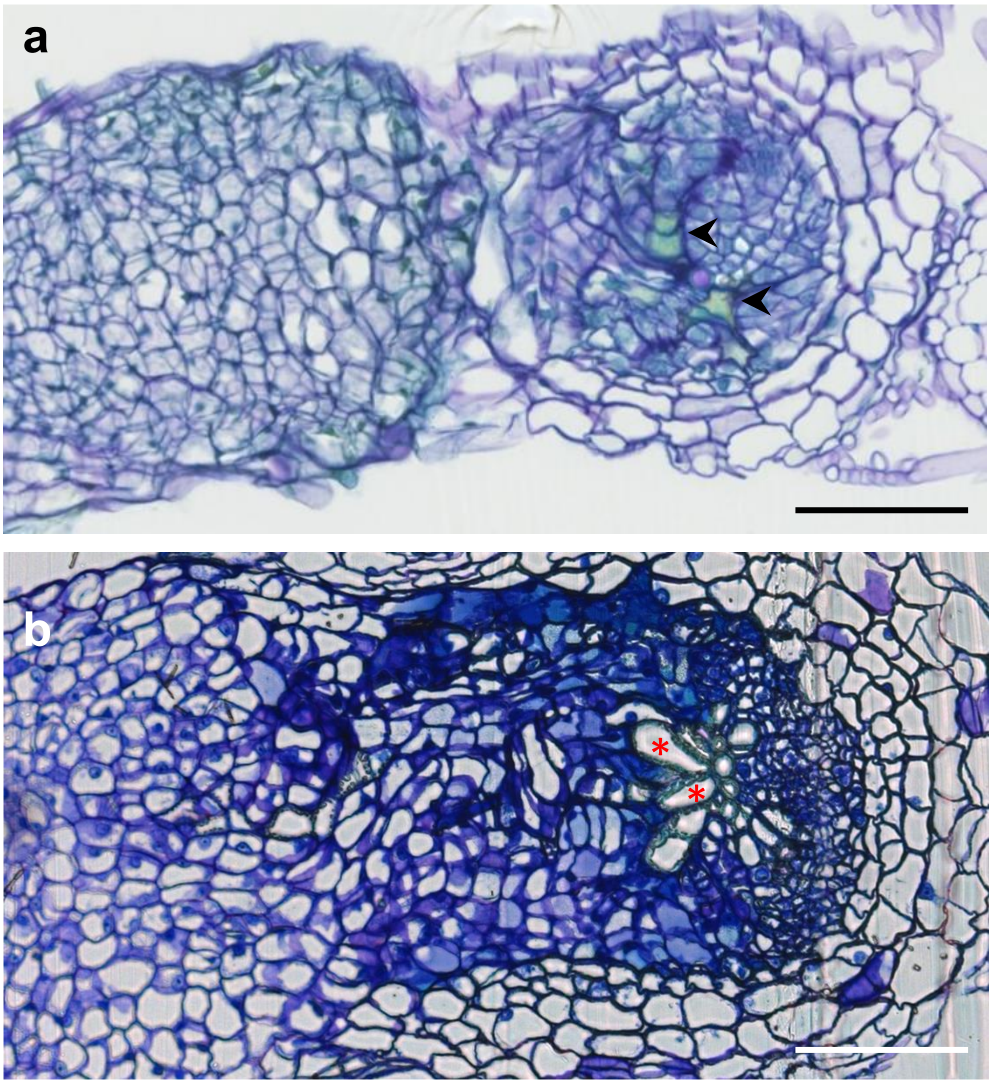

Suppl. File 13. Phenotyping in 6 L pots of wild *Helianthus* using the most virulent broomrape races G.

Boxplots of 7 accessions phenotyped in pots following inoculation with 4 races G (assay No 3) The accessions #2601 *H. exilis* and #325 *H. tuberosus* were from class I, #584 *H. bolanderi*, #677 *H. praecox*, #761 *H. petiolaris* and #786 *H. debilis tardiflorus* from class II and #833 *H. annuus* from class III. The 4 races G were from Romania (G-RO), Russia (G-RU), Spain (G-GV) and Turkey (G-TK). Five to 8 pots were cultured for each accession/ race. In each pot, the number of broomrape emergences was counted at the time of sunflower flowering. For each accession, Kruskal-Wallis test was performed (α=0.05). The p values were respectively: #2601ex: p=0.29; #325t (no p value); #584b: p=0.52; #677pr: p=0.12; #761p: p=0.004; #786d: p=0.03; #833a: p=0.06; B117: p=0.29; LP2: p=0.008.

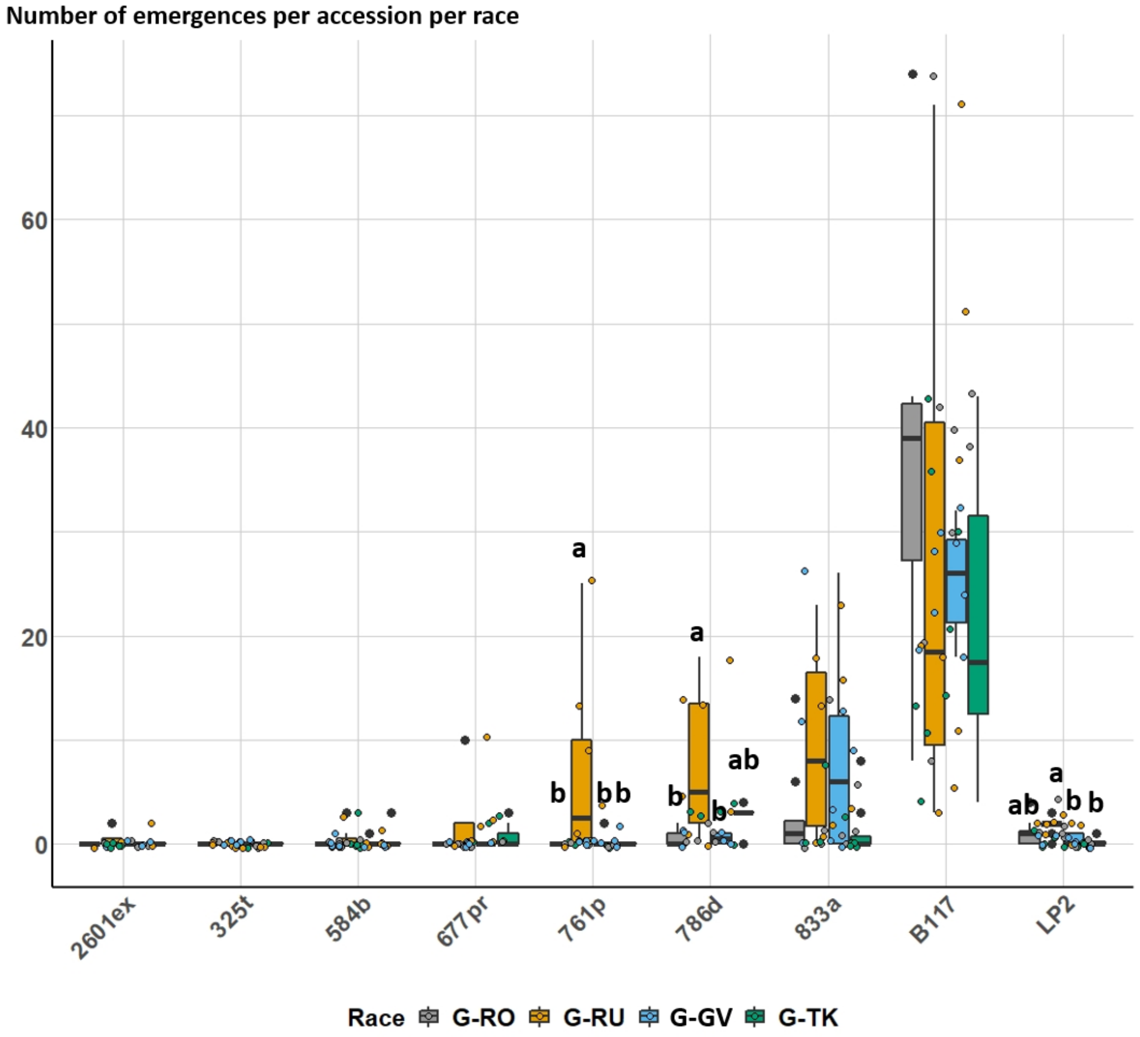

Supplement: Supplementary file 1 [file DataSheet_1.pdf]
